# Establishment and Validation of a Gene Signature-Based Prognostic Model to Improve Survival Prediction in Adrenocortical Carcinoma Patients

**DOI:** 10.1155/2021/2077633

**Published:** 2021-11-23

**Authors:** Xiaoqin Ge, Zhenzhen Liu, Xuehua Jiao, Xueyan Yin, Xiujie Wang, Gengxu Li

**Affiliations:** ^1^Department of Endocrinology, Suzhou Ninth Hospital Affiliated to Soochow University, Suzhou, China; ^2^Department of Endocrinology, Affiliated Hospital 2 of Nantong University and First People's Hospital of Nantong City, Nantong, China

## Abstract

**Background:**

The current guideline for the management of adrenocortical carcinoma (ACC) is insufficient for accurate risk prediction to guide adjuvant therapy. Given frequent and severe therapeutic side effects, a better estimate of survival is warranted for risk-specific assignment to adjuvant treatment. We attempted to construct an integrated model based on a prognostic gene signature and clinicopathological features to improve risk stratification and survival prediction in ACC.

**Methods:**

Using a series of bioinformatic and statistical approaches, a gene-expression signature was established and validated in two independent cohorts. By combining the signature with clinicopathological features, a decision tree was generated to improve risk stratification, and a nomogram was constructed to personalize risk prediction. Time-dependent receiver operating characteristic (tROC) and calibration analysis were performed to evaluate the predictive power and accuracy.

**Results:**

A three-gene signature could discriminate high-risk patients well in both training and validation cohorts. Multivariate regression analysis demonstrated the signature to be an independent predictor of overall survival. The decision tree could identify risk subgroups powerfully, and the nomogram showed high accuracy of survival prediction. Particularly, expression of a gene hitherto unknown to be dysregulated in ACC, TIGD1, was shown to be prognostically relevant.

**Conclusion:**

We propose a novel gene signature to guide decision-making about adjuvant therapy in ACC. The score shows unprecedented survival prediction and hence constitutes a huge step towards personalized management. As a secondary important finding, we report the discovery and validation of a new oncogene, TIGD1, which was consistently overexpressed in ACC. TIGD1 might shed further light on the biology of ACC and might give rise to targeted therapies that not only apply to ACC but potentially also to other malignancies.

## 1. Introduction

Adrenocortical carcinoma (ACC) is a rare but aggressive malignancy with a generally poor prognosis, with a 5-year overall survival (OS) rate less than 50% in most series [1–3]. The current tumor-node-metastasis (TNM) stage at diagnosis has been developed by using a large patient cohort from the European Network for the Study of Adrenal Tumors (ENSAT) [4] and independently validated [5]. However, variability in clinical outcomes even within the same tumor stage has stimulated the search for markers that harbor prognostic value. Histological tumor grade, resection status, expression of proliferation marker Ki-67, age, and symptoms (GRAS) have been shown to improve prognostication in advanced disease [6]. Molecular markers for an improved prognostication of ACC have been sought during recent years and models using targeted molecular marker assessment have been developed that could improve prediction of recurrence after complete resection but were of more limited value in ENSAT stage IV patients [7]. However, individual molecular markers have not yet changed treatment strategies in ACC [8, 9].

Transcriptome profiling has become possible with advancements of high-throughput techniques such as gene-expression microarray and RNA-sequencing (RNA-seq) and found widespread use in oncology research. It has yielded insights into changes of gene expression associated with malignancy in a variety of tumors at a global scale and demonstrated its potential for the discovery of diagnostic and prognostic biomarkers [10, 11].

Compared with single candidate biomarkers gene-expression signatures derived from multiple biomarkers may introduce less bias and increase the statistical power of the analysis. This strategy has therefore been applied to a number of different cancer types [12–15].

In this study, we mined public databases and developed a training data set to construct a risk score for ACC prognosis that was validated in two independent external cohorts from the ENSAT-consortium [16] and The Cancer Genome Atlas (TCGA) [17].

By inclusion of clinicopathological features, we aimed at improving predictive power and finally applied bioinformatic analyses to highlight biological processes and pathways underlying the newly discovered gene signature in ACC.

## 2. Materials and Methods

### 2.1. Data Set Preparation and Data Processing

All raw CEL files from two microarray data sets GSE10927 and GSE19750 measured on the same chip platform (Affymetrix HG-U133 Plus 2.0 Array, 54675 probes) were downloaded from GEO (http://www.ncbi.nlm.nih.gov/geo/) and were combined to a singular cohort using the robust multichip average (RMA) algorithm [18]. Of this singular cohort, samples with available clinical annotations and follow-up information were used as the training set. GSE10927 contains 33 ACC samples and 10 normal adrenal cortex samples, GSE19750 44 ACC samples, and 4 normal samples. For validation, 44 samples with follow-up information from GSE49278 (Affymetrix HG-2.0 ST Array, 53617 probes) were used as the first validation cohort. Probe IDs were mapped to gene symbols based on the corresponding annotation file, and expression measurements of all probes corresponding to the same gene were averaged to obtain a single value. Moreover, ACC samples from TCGA and normal samples from Genotype-Tissue Expression (GTEx) [19] database were obtained from UCSC Xena (https://xenabrowser.net/datapages/) to validate the expression profile and predictive value of the gene signature.

In addition, the somatic mutation profile of TCGA-ACC, which was identified using MuTect2, was sorted in the mutation annotation format (MAF) file. Using R package “maftools,” oncoplots were visualized based on the MAF files. Tumor mutation burden (TMB) was calculated with nonsynonymous somatic mutations using 38 Mb as the estimate of the exome size.

### 2.2. Candidate Selection and Signature Establishment

Firstly, normalized gene-expression profiles of 77 ACC samples and 14 normal tissues from the training set were used to screen for differentially expressed genes (DEGs) with “Limma” R package, and the argument of “adjust.method” was set as “fdr” for significance adjustment. DEGs were defined based on adjusted *p* values <0.01 and fold change (FC) <0.5 or >2. DEGs were subsequently studied by Cox regression analysis in the subcohort of 46 ACC patients with overall survival information available. Finally, a least absolute shrinkage and selection operator (LASSO) Cox regression model was used to further filter for the most robust prognostic markers in the training set. A risk score (RS) model was established by including individual normalized gene-expression values weighted by their LASSO Cox coefficients as follows:

(1) $$\begin{array}{c} \text{RS}=\sum_{i}\text{Coefficient}\left({\text{mRNA}}_{i}\right)\times \text{Expression}\left({\text{mRNA}}_{i}\right). \end{array}$$

Risk scores of each patient were calculated based on the above-mentioned formula.

### 2.3. Gene Co-Expression Network Analysis

The weighted gene co-expression network analysis (WGCNA) R package [20] was used to construct a scale-free co-expression network based on TCGA-ACC RNA-seq data. The weighed network adjacency *a* was defined as follows:

(2) $$\begin{array}{c} a_{i,j}=s_{i,j}^{\beta },\\ s_{i,j}=\left|\text{cor}\left(x_{i},x_{j}\right)\right|, \end{array}$$

where *i* and *j*: individual genes, *s*_*i*_: correlation; *x*_*i*_ and *x*_*j*_: gene-expression levels; cor: Pearson's correlation factor between two expression levels; and *β*: soft-power threshold. Adjacency was used to calculate the topological overlap matrix (TOM), and the corresponding dissimilarity (1 − TOM) was used as the distance measure with deepSplit of 2 and minModuleSize of 30 to generate different modules via hierarchical clustering analysis. Unassigned genes were categorized into the gray module. Among nongray modules, the module with the strongest correlation with risk score was selected for further study. All these steps were performed as previously reported [21]. Genes with GS > 0.4 involved in this module were submitted for Kyoto Encyclopedia of Genes and Genomes (KEGG) and Gene Ontology (GO) enrichment analysis. Circos was used for KEGG outputs visualization, and Metascape [22] was used for GO network visualization.

### 2.4. Statistical Analysis

IBM SPSS Statistics 20 (IBM Corp., Armonk, NY, USA), GraphPad Prism 8.0 (GraphPad Software Inc, San Diego, CA), Stata 12 (StataCorp LLC, Texas, USA), and R software (version 4.0.1, http://www.r-project.org) were used to analyze data and plot graphs. Kaplan–Meier method was used to construct survival curves, and differences were evaluated using the log-rank test. Cox proportional hazards regression model was applied to evaluate the significance of each variable for overall survival. Time-dependent receiver operating characteristic (tROC) analysis was used to measure the predictive power with “survivalROC” package, and areas under the curve (AUC) of each variable at different time nodes were compared. Meta-analysis was performed to evaluate the prognostic value in the pooled cohort. Risk scores in each cohort were scaled to Z-scores. Recursive partitioning analysis (RPA) was performed to construct decision trees to identify different risk subgroups using “rpart” package. Nomogram and calibration curve were plotted using “rms” package. Student's *t*-test or one-way ANOVA was used to analyze differences.

## 3. Results

### 3.1. Establishment of a Prognostic Gene Signature in a Training Set

**Figure 1:**
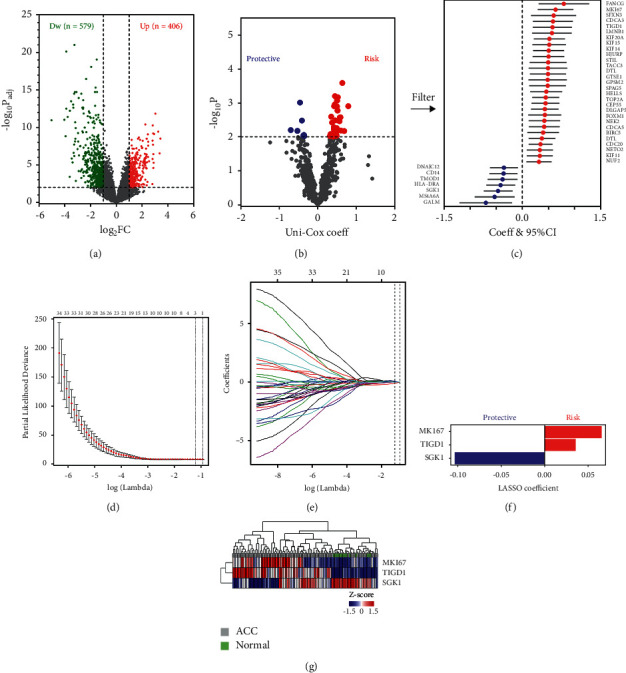
Selection of robust biomarkers to establish a survival-related gene signature: (a) a total of 985 DEGs were identified in ACC compared to normal tissues; (b and c) 35 promising candidates were filtered out using univariate Cox regression analysis; (d) cross-validation was applied to prevent overfitting, and the optimal *λ* value of 0.2967 with log(*λ*) = −1.215 was selected; (e) MKI67, TIGD1, and SGK1 finally remained with their nonzero LASSO coefficients; (f) distribution of LASSO coefficients of the gene signature; and (g) hierarchical clustering analysis showed normal tissues were characterized by lower expression levels of MKI67 and TIGD1 and by higher SGK1 expression levels compared to ACC.

In the training set, a total of 985 DEGs (579 downregulated and 406 upregulated) were identified in 77 ACC samples compared to 14 normal samples (Figure 1(a)). Cox regression identified 35 DEGs with prognostic relevance (7 protective markers and 28 risk markers; Figure 1(b)). In Figure 1(c), univariate Cox coefficients and 95% confidence intervals (CI) are plotted. Cross-validation was applied to prevent the overfitting of the LASSO Cox model, and the optimal *λ* value of 0.2967 with log(*λ*) = −1.215 was selected (Figure 1(d)). As shown in Figures 1(e) and 1(f), three genes (MKI67, TIGD1, and SGK1) finally remained with their individual nonzero LASSO Cox coefficients. Hierarchical clustering analysis indicated that normal tissues were characterized by lower expression levels of MKI67 and TIGD1 and higher expression levels of SGK1 compared to ACC tissues in the training set (Figure 1(g)). Gene-expression levels in ACC and normal tissues in the combined GEO cohorts are shown in Supplementary Figure 1.

### 3.2. Gene Signature Serves as a Risk Factor and Promising Predictor for OS

**Figure 2:**
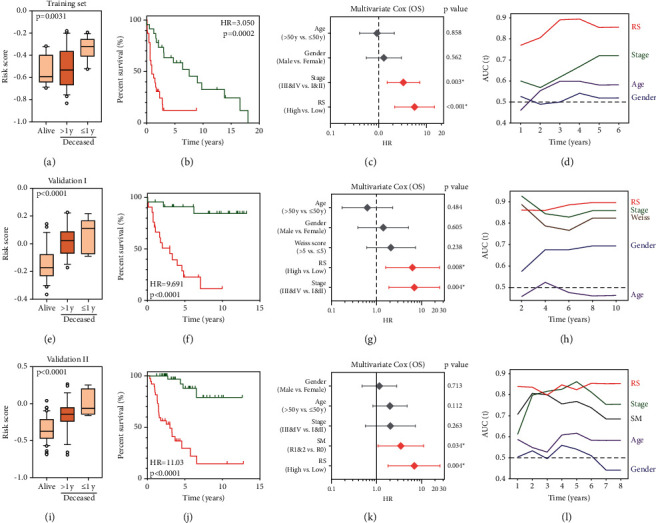
Gene signature-derived risk score serves as a risk factor and reliable predictor for overall survival in each cohort. (a–d) In the training cohort, the signature-based risk score was significantly elevated in patients who died during follow-up, especially in the short-term survival group. Kaplan–Meier survival analysis demonstrated a worse prognosis in patients with a high-risk score. Multivariate Cox regression analysis showed that risk score and TNM stage were independent risk factors for overall survival. At different time points, tROC analysis showed risk score was an accurate predictor for survival with even better performance than TNM stage. (e–h) Validation cohort I. (i–l) Validation cohort II.

Compared to 128 GTEx normal tissues, RSEM-normalized counts of MKI67 and TIGD1 mRNA were significantly upregulated in TCGA ACC, while SGK1 was significantly downregulated (Supplementary Figure 2). With the formula of risk score = (0.036112 ∗ expression level of TIGD1) + (−0.10388 ∗ expression level of SGK1) + (0.065722 ∗ expression level of MKI67), we calculated the risk scores for all the patients involved in our study. In the training cohort, compared to alive patients, the risk score was significantly elevated in patients who died during follow-up, especially in short-term survival patients who died within 1 year after surgery (Figure 2(a)). Kaplan–Meier analysis revealed that patients with higher risk scores exhibited worse prognosis than those with lower scores (HR = 3.050, 95% CI = 1.459–6.378, and *p* = 0.0002; Figure 2(b)). Subsequently, multivariate Cox regression modeling demonstrated that AJCC TNM stage (HR = 3.272, 95% CI = 1.495–7.159, and *p* = 0.003) and risk score (HR = 5.580, 95% CI = 2.143–14.53, and *p* < 0.001) are independent, statistically significant risk factors for overall survival (OS) in the training cohort (Figure 2(c)). tROC analysis showed that the gene-expression signature-derived risk score was an accurate predictor for OS and performed better than the traditional TNM stage, while age and gender exhibited little predictive power (Figure 2(d)).

To confirm the prognostic robustness of the gene signature in different series, it was further validated in two independent external cohorts: GSE49278 (validation cohort I, Figures 2(e)–2(h)) and TCGA (validation cohort II, Figures 2(i)–2(l). Similarly, in the two validation cohorts, compared to alive patients, the risk score was significantly elevated in patients who died during follow-up; again in short term, survival patients were characterized by high-risk scores. Kaplan–Meier analysis demonstrated that patients with higher risk scores exhibited worse OS (validation I: HR = 9.691, 95% CI = 3.787–24.80, and *p* < 0.0001 and validation II: HR = 11.03, 95% CI = 5.079–23.95, and *p* < 0.0001). Furthermore, multivariate Cox regression analysis was performed on risk score together with clinicopathological variables, including age, gender, Weiss score, and ENSAT stage in validation cohort I, while age, gender, surgical margin (SM: R0, R1, and R2), and AJCC stage were used in validation cohort II. Like in the training cohort, the risk score was independently associated with OS in both validation cohorts (validation I: HR = 6.280, 95% CI = 1.606–24.561, and *p* = 0.008 and validation II: HR = 7.082, 95% CI = 1.856–27.03, and *p* = 0.004). The risk score outperformed other clinicopathological variables in terms of predictive accuracy in tROC analysis with an average AUC above 0.8 in the follow-up period in the two validation cohorts.

**Figure 3:**
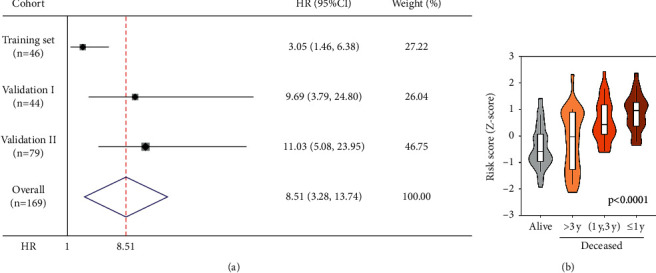
Gene signature-derived risk scores could discriminate high-risk patients in the pooled cohort: (a) meta-analysis indicated patients with higher risk scores exhibited worse overall survival with an integrated HR of 8.51 (95% CI = 3.28–13.74) and (b) Z-scores of risk score were significantly elevated in patients who died during follow-up, especially in shorter-term survival groups (*p* < 0.0001).

The combination of all three cohorts revealed that an elevated risk score conferred an HR of 8.51 (95% CI = 3.28–13.74) over patients with a lower score (Figure 3(a)). Additionally, in the pooled cohort, Z-transformed risk scores were significantly elevated in those patients who died during follow-up, with progressively increasing Z-scores as survival time decreased (Figure 3(b)). Moreover, when patients were stratified by gender, age, TNM stage, and R status, the risk score retained its predictive capability, thereby precluding that the aforementioned variables act as confounders (Supplementary Figure 3). The sole exemption was the small subgroup of patients with positive resection status (R1 and R2).

### 3.3. Combination with Clinical Variables to Improve Risk Stratification and Survival Prediction

**Figure 4:**
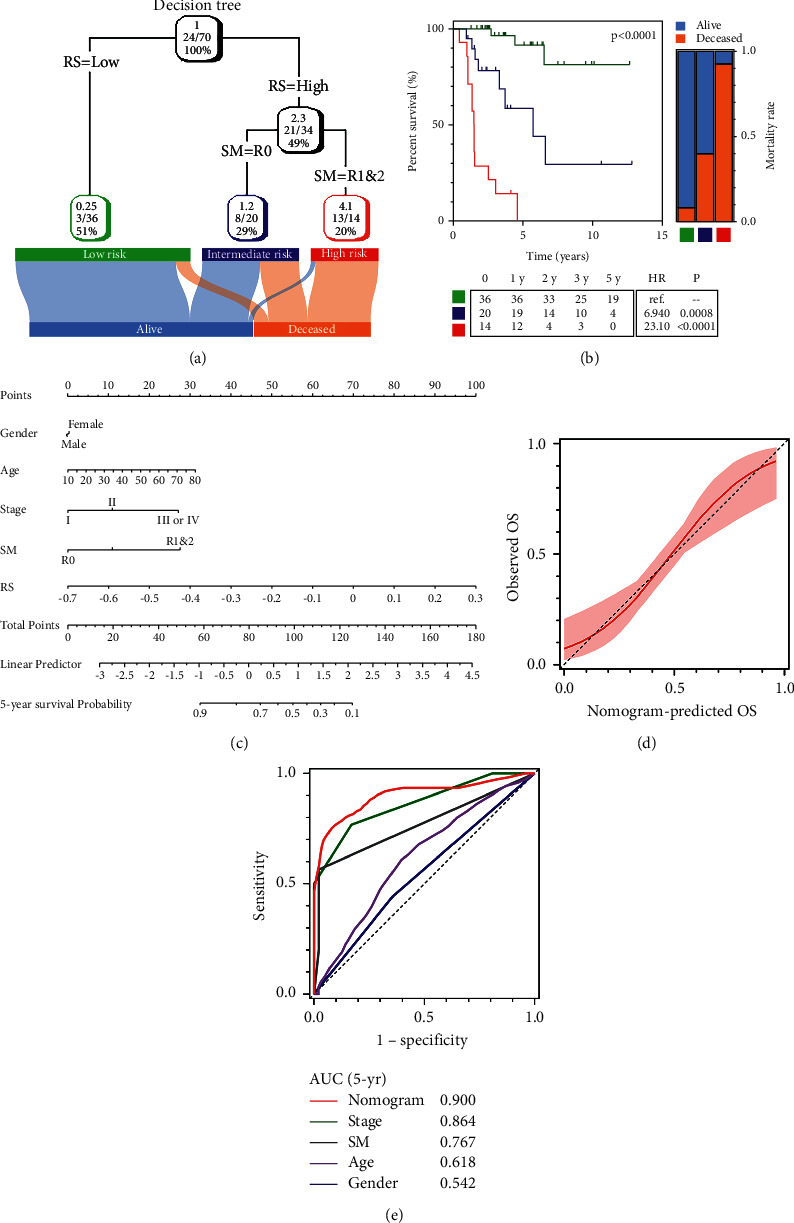
Construction of an integrated model to optimize risk stratification and personalize risk assessment: (a) a decision tree was generated based on the TCGA cohort, with a risk score and SM status incorporated as two major components; (b) overall survival differed significantly in different risk subgroups, and mortality increased gradually as risk level increased; (c) a nomogram was constructed to personalize risk assessment for individual patients; (d) the calibration curve showed the nomogram-predicted survival to be close to ideal performance; and (e) tROC analysis of all clinicopathological parameters demonstrated the greatest accuracy of the nomogram for survival prediction.

Recursive partitioning analysis (RPA) was performed to construct a decision tree to improve risk stratification for survival based on the TCGA cohort as clinical annotations of the patients in this data set were most comprehensive. Five parameters including age, gender, AJCC TNM stage, resection status (R0 or R1 and R2), and risk score were submitted to construct the decision tree. Three different risk subgroups (“low-risk,” “intermediate-risk,” and “high-risk”) were derived for overall survival based on the two major parameters including risk score as the most powerful component together with R status (Figure 4(a)). As shown by the Kaplan–Meier plot in Figure 4(b), the three risk subgroups differed markedly in overall survival. The subgroup labeled with high risk showed the highest mortality rate (92.9%) and most unfavorable outcome (HR = 23.10, 95% CI = 6.027–88.52, and *p* < 0.0001) compared to the low-risk subgroup.

With the goal of a quantitative model to predict the 5-year survival probability for individual patients after surgery, a nomogram was built with a risk score and other clinicopathological variables as retrieved from the TCGA cohort (Figure 4(c)). In the calibration analysis, the predictive line of the nomogram (red) was extremely close to the ideal 45-degree line (dotted), indicating the good performance of the nomogram (Figure 4(d)). Compared with the other variables, the nomogram score exhibited the most accurate prediction power with an AUC of 0.900 (Figure 4(e)).

### 3.4. Putative Biological Processes Underlying the Gene Signature as Derived from Bioinformatics

**Figure 5:**
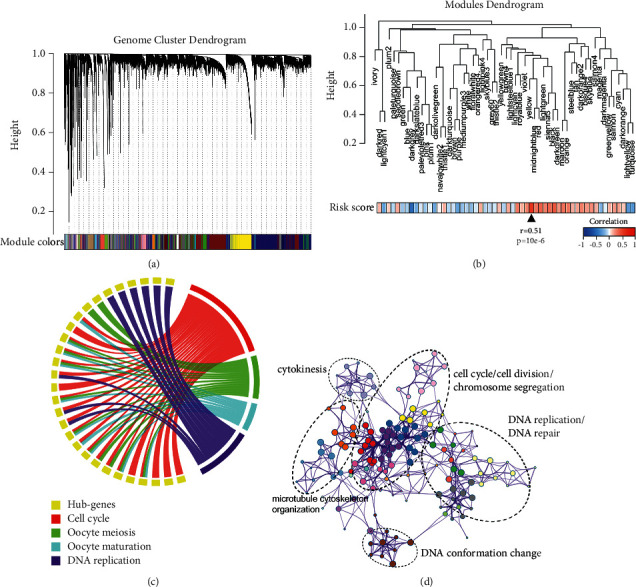
Bioinformatics analyses were performed for annotations of biological processes underlying the gene signature: (a) whole-genome cluster dendrogram; (b) modules dendrogram and heatmap of relationships between risk score and different modules; (c) Circos showed that most hub genes were enriched in the cell-cycle-related processes; and (d) visualized GO enrichment biological network.

No outlier was detected after sample clustering (Supplemental Figure 4). A total of 79 TCGA-ACC samples with corresponding risk scores were used to construct a weighted gene co-expression network. A power of *β* = 5 (scale-free *R*^2^ = 0.9) was set as the optimal soft threshold to ensure a scale-free network (Supplemental Figure 5). Cluster dendrogram trees of the whole genome were constructed, and a total of 57 modules were generated (Figure 5(a)).  Figure 5(b) shows the modules dendrogram and the heatmap of relationships between risk score and different modules, with the yellow module depicting the highest correlation (*r* = 0.51 and *p* = 1*e* − 06). With a threshold of GS > 0.4, hub genes extracted from the yellow module (Supplementary Table 1) were submitted for enrichment analysis. Circos plot showed that hub genes were mainly enriched in cell-cycle-related processes using KEGG analysis (Figure 5(c)). Moreover, GO enrichment analysis showed an association of the main parts of the biological network with “cell division,” “cell cycle,” “DNA replication,” “microtubule cytoskeleton organization,” and so on (Figure 5(d)). Of note, a number of genes that were reportedly co-expressed with TIGD1 in hepatocellular carcinoma—as determined by in silico analysis—could also be found among the hub genes associated with the risk score. The associated processes encompass homologous recombination, DNA replication, and cell cycle progression. Likewise, we found further biomarkers such as CDK1 and BUB1B that have previously been reported to be differentially expressed in ACC to appear in the yellow module, thereby validating our findings.

### 3.5. Mutational Analysis in Different Risk Score Groups

**Figure 6:**
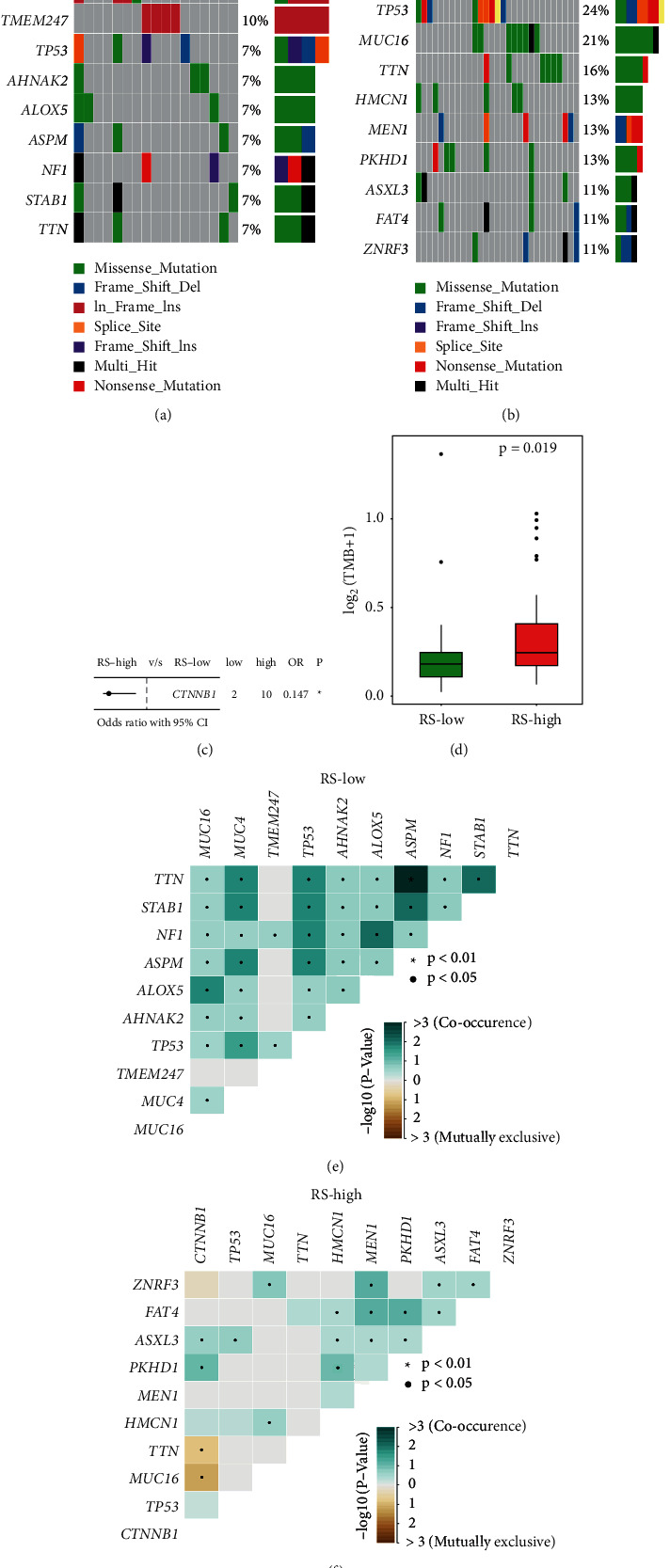
Mutational analysis in different risk groups: (a and b) oncoplots for ACC samples with low- and high-risk scores were generated, respectively; (c) with a threshold of *p* < 0.05 using Fisher's exact test, a differentially mutated gene named CTNNB1 was detected between the two risk cohorts; (d) significant higher tumor mutation burden (TMB) was observed in ACC samples with higher risk scores; and (e and f) more co-occurrence mutations were observed in the low-RS cohort, and more mutually exclusive mutations were observed in the high-RS cohort.

Oncoplots for ACC samples with low- and high-risk scores were generated. In the low-risk cohort, MUC16, MUC4, TMEM247, and TP53 exhibited a mutation frequency of 10% (Figure 6(a)). In contrast, TP53 exhibited a much higher mutation frequency of 24% in the high-risk cohort (Figure 6(b)). With a threshold of *p* < 0.05 using Fisher's exact test, a differentially mutated gene named CTNNB1 was detected between the two risk cohorts (Figure 6(c)). In addition, a significant higher tumor mutation burden (TMB) was observed in ACC samples with higher risk scores (*p* = 0.019; Figure 6(d)). With regard to mutational features, more co-occurrence mutations were observed in the low-RS cohort, and more mutually exclusive mutations were observed in the high-RS cohort (Figures 6(e) and 6(f)).

## 4. Discussion

Adrenocortical cancer is a highly invasive and challenging malignancy with poor overall survival, with recurrence rates even after R0 resection being as high as 72%. Moreover, deciding which patients will benefit from adjuvant therapy is not possible at present. The side-effect profile of mitotane—the only drug approved for adjuvant therapy in ACC—is substantial and very often has a profound negative impact on quality of life. Guidelines currently recommend to rather initiate therapy in patients with a high risk of recurrence but acknowledge that this recommendation is based on weak evidence. No recommendations are made for patients with low or intermediate risk. Therefore, the development of reliable markers for survival prediction would greatly optimize risk stratification and improve the management of ACC. Currently, the staging system proposed by the European Network for the Study of Adrenal Tumors (ENSAT) is recommended and widely used in clinical routines. It mainly relies on the traditional TNM classification [23]. However, its predictive power and accuracy are often insufficient, and for clinical outcomes, it may exhibit considerable variation especially in localized ACC stages.

In recent years, microarray profiling and RNA-sequencing techniques have received increasing attention with the extraction of diagnostic and prognostic biomarkers through the analysis of thousands of dysregulated genes in malignancy. However, until now, only few studies have focused on survival prediction in ACC. In this study, we established a three-gene signature combined with clinicopathological variables to construct an integrated model to offer precise risk stratification and overall survival prediction for patients with ACC.

We used publicly available expression data sets and could show three genes to be dysregulated in ACC: MKI67, TIGD1, and SGK1. As for TIGD1, this is the first report of this gene to be upregulated in ACC. Two reasons may account for the fact that we were able to extract a new biomarker from the above-mentioned analyzed cohorts: to the best of our knowledge, our group is the first one to combine the two GEO data sets GSE10927 and GSE19750 (homogeneity being ensured by the fact that both data sets were derived from the same platform). The resulting unique discovery cohort allowed us to detect TIGD1 expression as upregulated in ACC. Other groups integrated the TCGA cohort and the ENSAT cohort into a common discovery cohort [7], or they combined GEO data sets GSE12368 and GSE19750 [24] or focused exclusively on the TCGA data set [25].

Another reason that may have allowed TIGD1 to be detected for the first time in the context of ACC may be the use of the LASSO method to prevent overfitting.

TIGD1 was first identified as a member of the tigger subfamily of the pogo superfamily of DNA-mediated transposons in humans [26]. Recently, TIGD1 was predicted as a cell-cycle-related biomarker upregulated in various cancer types and indicated a worse prognosis in these cases [27]. Moreover, the response of ovarian cancer to platinum-based chemotherapy has been linked to TIGD1 [28]. Given that platinum is an integral component of the EDP-M regimen for chemotherapy of ACC [29], these findings are of potential applicability in patients with ACC amenable to cytotoxic chemotherapy. It is further tempting to speculate on the oncogenic potential of TIGD1 as a DNA transposon, but this requires prospective investigation in easily accessible models such as cell culture lines to elucidate both pathophysiological role and potential clinical significance since this is clearly not an established transposon oncogene like LINE-1 [30].

In adrenal specimen, immunohistochemistry for Ki67 yields the Ki67 index that is used next to histopathological scoring systems like the Weiss and the Helsinki score to establish a diagnosis of malignancy [23]. Aside from its diagnostic capacities, the Ki67 index has been validated as a reliable and powerful parameter to predict survival in ACC patients after complete tumor resection [31]. In the light of this body of literature, we perceive the extraction of MKI67 as independent validation of our strict selection criteria.

In our gene-expression signature, SGK1 showed characteristics of a protective factor (tumor suppressor gene). Ronchi et al. reported low SGK1 expression to be associated with poor overall survival in ACC patients independent of tumor stage [32], which is consistent with our findings of the putative nature of SGK1 in this context. The authors of the latter publication speculated on disinhibition of the Notch signaling pathway by reduced SGK1 expression. Since a subgroup of patients with high *β*-catenin scores and low SGK1 expression exhibited the worst prognosis—albeit under these conditions SGK1 should be positively regulated by *β*-catenin—this axis was interpreted to be disrupted [32].

Explorative enrichment analysis of our gene signature revealed that hub genes were enriched in cell-cycle-related processes. We also observed the core parts in the GO enrichment network were labeled with “cell cycle,” “cell division,” “DNA replication,” and “microtubule cytoskeleton organization.” The specific labeling of core components in the GO enrichment network analysis suggested that the gene signature might contribute to poor survival in ACC via increased proliferative activity [33].

From the three above-described differentially expressed genes, we were able to derive a risk score, which correlated well with overall survival. Confounding of gender, age, and tumor stage was excluded. Interestingly, we could validate the risk score in two independent external risk cohorts and construct a meta-analysis from the three studies. Moreover, multivariate Cox regression analysis indicated the signature-derived risk score, advanced TNM stage, and positive surgical margin are closely correlated with poor survival. In each cohort, tROC analysis revealed the risk score to exhibit the highest predictive accuracy for 5-year overall survival, the accuracy being greater than traditional TNM staging. In the pooled cohort, when stratified by important clinicopathological variables, the gene signature could discriminate high-risk patients powerfully, supporting its value as an independent predictor in different subgroups.

A decision tree was generated to improve risk stratification in combination with clinicopathological features. In the decision tree, the risk score served as the major determinant, superior to any other conventional factor. When stratified by a decision tree, overall survival varied dramatically in the resulting risk subgroups. A nomogram was constructed to quantify risk for individual ACC patients including the risk score with other clinicopathological features based on the TCGA cohort. tROC analysis showed that the nomogram exhibited a more powerful and accurate prediction than any other single variable, with the AUC(t) of 5-year survival prediction amounting to 0.900.

The model proposed in the present study on the other hand incorporates traditional clinicopathological characteristics as well as individual tumor biology to arrive at a comprehensive prediction tool for 5-year overall survival in ACC.

The mutational features in different risk cohorts were analyzed and compared. We observed that more co-occurrence mutations were observed in the low-risk cohort, and more mutually exclusive mutations were observed in the high-risk cohort. In addition, TMB was significantly higher in the high-risk cohort compared to the low-risk cohort, which suggests that ACCs with a higher risk score might exhibit higher tumor heterogeneity.

Some limitations of our study should be acknowledged: Firstly, this is a retrospective study with limited sample size, so the robustness and clinical usefulness of the gene signature should be further validated in larger prospective clinical trials. A feasible approach might consist of a validation of the expression levels by quantitative real-time PCR with appropriate reference genes for normalization purposes. After validation, this method can be used in a prospective trial. This technique is already employed in assays such as the Oncotype DX test to predict the likelihood of recurrence in breast cancer and guide decision-making about adjuvant chemotherapy. Based on the outcomes of the prospective TAILORx trial, the American Society of Clinical Oncology recently issued an update outlining when physicians should recommend for or against chemoendocrine therapy with a given Oncotype DX risk score in patients with early-stage invasive breast cancer [34]. If we could promote a comparable evolution of therapeutic pathways in ACC, a lot of harm might be prevented, and we might serve our patients better. Secondly, further experimental studies are required to elucidate the dysregulated pathways underlying the combined alterations resulting from the gene signature in ACC. Particular interest should be paid to the biological role of TIGD1 and its putative nature as a transposon. Clarification of the systems biology network of TIGD1 will likely foster the development of novel therapeutic strategies for the treatment of ACC.

In summary, we established a novel gene-expression signature to predict overall survival in ACC. Combined with clinicopathological features, improved stratification allows for individualized risk quantification in patients. We hope this integrated model will prove to be a useful tool for future personalized management of patients with ACC.

## Data Availability

This study is based on published or public data sets and does not include new data that require ethical approval and consent.

## Supplementary Materials

Supplementary Materials Supplementary Figure 1: Boxplots depict the significant differences in expression patterns between ACC and normal tissues in the training cohort. Supplementary Figure 2: Boxplots depict the significant differences in expression patterns between TCGA-ACC and GTEx normal tissues. Supplementary Figure 3: In the pooled cohort, the risk score serves as a valuable marker to predict overall survival in differently stratified subgroups, including stratifications by gender, age, TNM stage, and SM subgroups. Supplementary Figure 4: no outlier was detected after sample clustering. Supplementary Figure 5: the power of *β* = 5 (scale-free *R*^2^ = 0.9) was set as the optimal soft threshold to ensure a scale-free network. Supplementary Table 1: hub genes extracted from the yellow module.
