# Supplementary material for: Establishment and Validation of a Gene Signature-Based Prognostic Model to Improve Survival Prediction in Adrenocortical Carcinoma Patients
Source: Int J Endocrinol. 2021 Nov 23;2021:2077633. doi: 10.1155/2021/2077633 (PMC8632466; doi:10.1155/2021/2077633)
Supplement: Supplementary Materials — Supplementary Figure 1: Boxplots depict the significant differences in expression patterns between ACC and normal tissues in the training cohort. Supplementary Figure 2: Boxplots depict the significant differences in expression patterns between TCGA-ACC and GTEx normal tissues. Supplementary Figure 3: In the pooled cohort, the risk score serves as a valuable marker to predict overall survival in differently stratified subgroups, including stratifications by gender, age, TNM stage, and SM subgroups. Supplementary Figure 4: no outlier was detected after sample clustering. Supplementary Figure 5: the power of β = 5 (scale-free R2 = 0.9) was set as the optimal soft threshold to ensure a scale-free network. Supplementary Table 1: hub genes extracted from the yellow module. [file 2077633.f1.docx]

**Supplementary figures and table**

Supplementary figure 1. Boxplots depict the significant differences in expression patterns between ACC and normal tissues in the training cohort.

Supplementary figure 2. Boxplots depict the significant differences in expression patterns between TCGA-ACC and GTEx normal tissues.

Supplementary figure 3. In the pooled cohort, the risk score serves as a valuable marker to predict overall survival in differently stratified subgroups, including stratifications by gender, age, TNM stage and SM subgroups.

Supplementary figure 4. No outlier was detected after sample clustering.

Supplementary figure 5. The power of β = 5 (scale-free R^2^ = 0.9) was set as the optimal soft threshold to ensure a scale-free network.

Supplementary Table 1. Hub Genes extracted from the yellow module

Suppl Figure 1


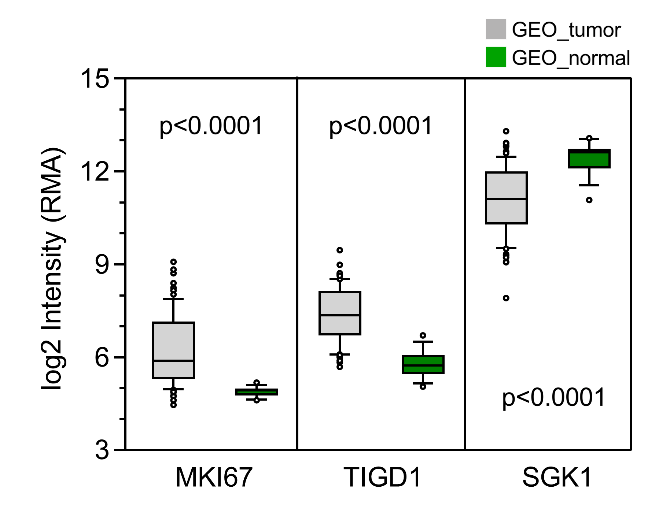


Suppl Figure 2


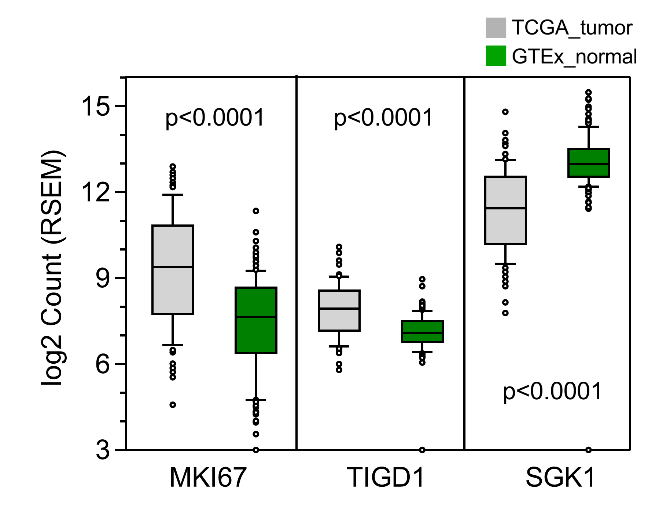


Suppl Figure 3


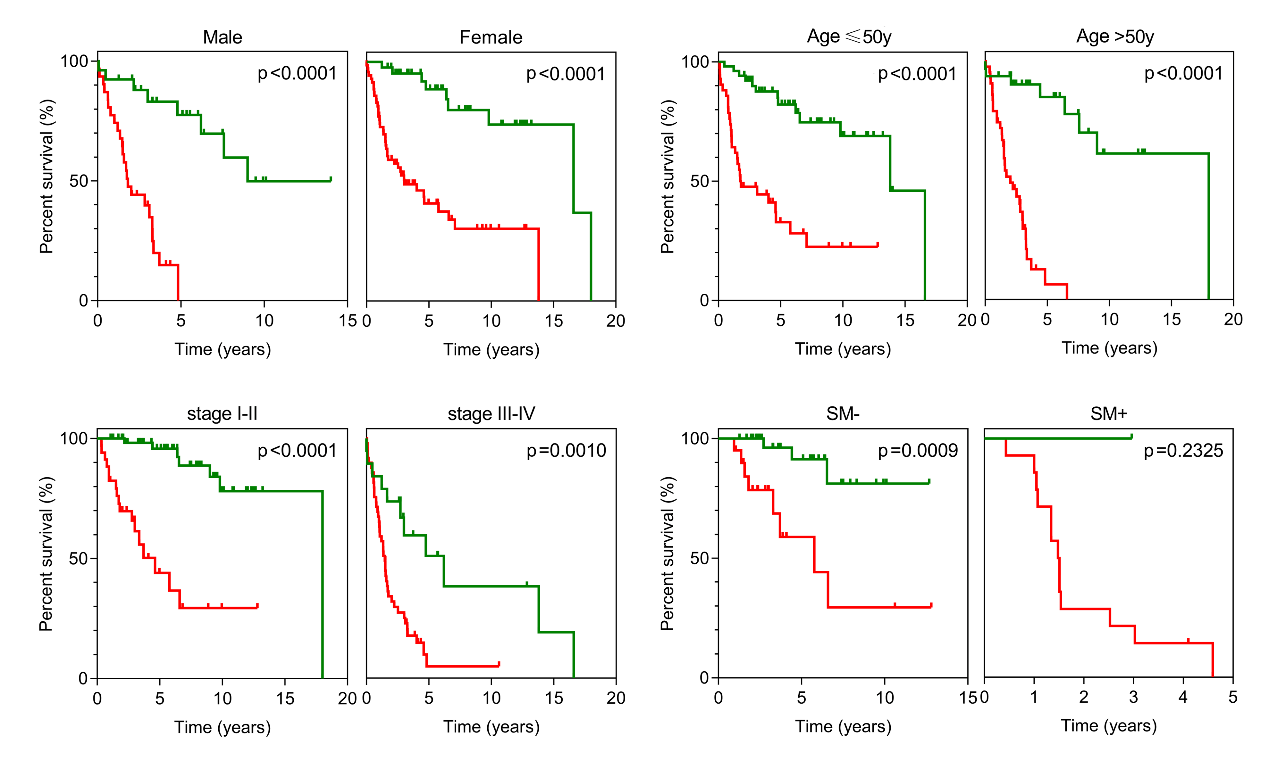


Suppl Figure 4


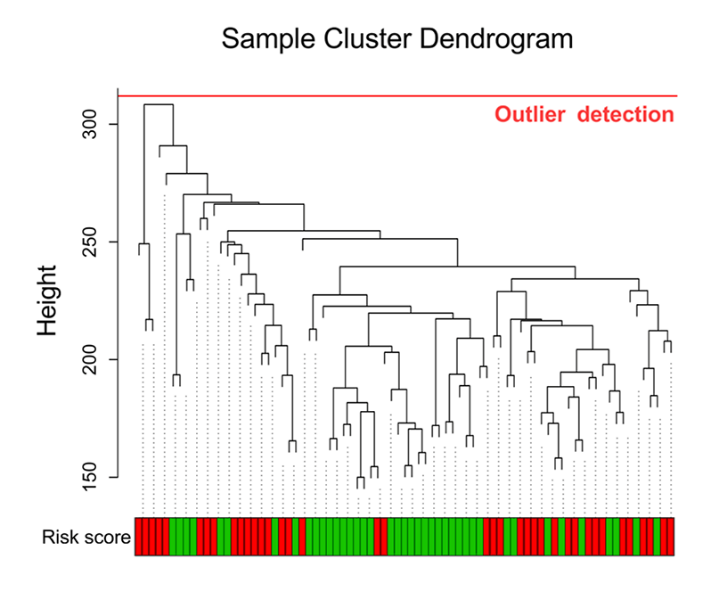


Suppl Figure 5


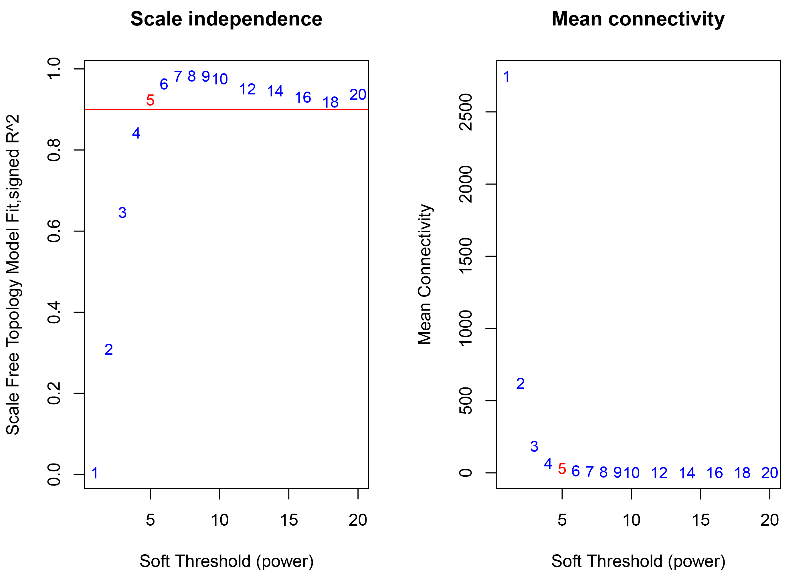


Supplementary Table 1. Hub Genes extracted from the yellow module

| **Genes** | **GS.RS** | **p.GS.RS** | **Module** |
| --- | --- | --- | --- |
| NDC80 | 0.668401 | 1.69E-11 | yellow |
| PSMC3IP | 0.650877 | 8.38E-11 | yellow |
| CDCA3 | 0.627014 | 6.32E-10 | yellow |
| RHEBL1 | 0.608937 | 2.61E-09 | yellow |
| AURKB | 0.60758 | 2.90E-09 | yellow |
| C9orf100 | 0.604247 | 3.72E-09 | yellow |
| CDC20 | 0.598117 | 5.86E-09 | yellow |
| ASPM | 0.597712 | 6.04E-09 | yellow |
| MKI67 | 0.595419 | 7.13E-09 | yellow |
| TROAP | 0.595286 | 7.20E-09 | yellow |
| SPC25 | 0.581645 | 1.90E-08 | yellow |
| GTSE1 | 0.581575 | 1.90E-08 | yellow |
| HJURP | 0.579447 | 2.21E-08 | yellow |
| MXD3 | 0.5786 | 2.34E-08 | yellow |
| UBE2C | 0.577736 | 2.48E-08 | yellow |
| CDC45 | 0.57361 | 3.28E-08 | yellow |
| GPC2 | 0.571341 | 3.82E-08 | yellow |
| CEP55 | 0.569963 | 4.19E-08 | yellow |
| DNAJC9 | 0.569219 | 4.40E-08 | yellow |
| CDK1 | 0.568175 | 4.72E-08 | yellow |
| KIF18B | 0.567524 | 4.93E-08 | yellow |
| UHRF1 | 0.564285 | 6.10E-08 | yellow |
| H2AFX | 0.554853 | 1.12E-07 | yellow |
| PLK1 | 0.553138 | 1.25E-07 | yellow |
| PIF1 | 0.553029 | 1.26E-07 | yellow |
| TOP2A | 0.552925 | 1.26E-07 | yellow |
| KIF20A | 0.550126 | 1.51E-07 | yellow |
| NCAPG | 0.549834 | 1.53E-07 | yellow |
| KIFC1 | 0.547817 | 1.74E-07 | yellow |
| CCNA2 | 0.546971 | 1.83E-07 | yellow |
| CENPF | 0.546697 | 1.86E-07 | yellow |
| C16orf75 | 0.545308 | 2.03E-07 | yellow |
| CENPM | 0.544728 | 2.10E-07 | yellow |
| PTTG1 | 0.544488 | 2.14E-07 | yellow |
| KIF14 | 0.542148 | 2.46E-07 | yellow |
| MCM10 | 0.541193 | 2.61E-07 | yellow |
| EME1 | 0.539982 | 2.81E-07 | yellow |
| MELK | 0.536308 | 3.50E-07 | yellow |
| STMN1 | 0.535859 | 3.60E-07 | yellow |
| SPAG5 | 0.53331 | 4.19E-07 | yellow |
| MYBL2 | 0.532999 | 4.26E-07 | yellow |
| C14orf80 | 0.532316 | 4.44E-07 | yellow |
| BUB1 | 0.530966 | 4.81E-07 | yellow |
| NUF2 | 0.530311 | 4.99E-07 | yellow |
| PRC1 | 0.530262 | 5.01E-07 | yellow |
| NEK2 | 0.52981 | 5.14E-07 | yellow |
| DLGAP5 | 0.528962 | 5.40E-07 | yellow |
| IQGAP3 | 0.528658 | 5.50E-07 | yellow |
| DNA2 | 0.528238 | 5.64E-07 | yellow |
| PHF19 | 0.526517 | 6.23E-07 | yellow |
| CENPA | 0.526409 | 6.27E-07 | yellow |
| LEPRE1 | 0.526034 | 6.40E-07 | yellow |
| DONSON | 0.525406 | 6.64E-07 | yellow |
| PTTG3P | 0.524367 | 7.05E-07 | yellow |
| CDT1 | 0.523471 | 7.42E-07 | yellow |
| LMNB1 | 0.523203 | 7.53E-07 | yellow |
| CDC7 | 0.522614 | 7.79E-07 | yellow |
| RECQL4 | 0.522507 | 7.84E-07 | yellow |
| CCNB1 | 0.520719 | 8.68E-07 | yellow |
| KIAA0101 | 0.52031 | 8.89E-07 | yellow |
| HAUS8 | 0.5194 | 9.35E-07 | yellow |
| KIF11 | 0.518592 | 9.79E-07 | yellow |
| GSG2 | 0.518361 | 9.92E-07 | yellow |
| HELLS | 0.516841 | 1.08E-06 | yellow |
| AURKAPS1 | 0.516086 | 1.13E-06 | yellow |
| C16orf59 | 0.512067 | 1.41E-06 | yellow |
| CDCA8 | 0.511906 | 1.42E-06 | yellow |
| UBE2T | 0.511681 | 1.44E-06 | yellow |
| CDKN3 | 0.511268 | 1.47E-06 | yellow |
| CDCA2 | 0.510735 | 1.52E-06 | yellow |
| FANCC | 0.509843 | 1.59E-06 | yellow |
| KAZALD1 | 0.509169 | 1.65E-06 | yellow |
| FANCG | 0.50906 | 1.66E-06 | yellow |
| KIF4A | 0.508149 | 1.75E-06 | yellow |
| CNTROB | 0.507138 | 1.85E-06 | yellow |
| LMNB2 | 0.506465 | 1.92E-06 | yellow |
| EXO1 | 0.505177 | 2.05E-06 | yellow |
| HIST1H3C | 0.504908 | 2.08E-06 | yellow |
| TRIP13 | 0.503969 | 2.19E-06 | yellow |
| FAM72B | 0.502784 | 2.34E-06 | yellow |
| C15orf42 | 0.50247 | 2.38E-06 | yellow |
| KIF18A | 0.501859 | 2.45E-06 | yellow |
| PKMYT1 | 0.50159 | 2.49E-06 | yellow |
| CDCA5 | 0.50122 | 2.54E-06 | yellow |
| TACC3 | 0.500859 | 2.59E-06 | yellow |
| AURKA | 0.500538 | 2.63E-06 | yellow |
| DDX39 | 0.500409 | 2.65E-06 | yellow |
| KIF23 | 0.497546 | 3.09E-06 | yellow |
| MLF1IP | 0.497292 | 3.13E-06 | yellow |
| KIF2C | 0.495327 | 3.47E-06 | yellow |
| WDR62 | 0.494044 | 3.71E-06 | yellow |
| CCNB2 | 0.493607 | 3.79E-06 | yellow |
| KCTD19 | 0.492963 | 3.92E-06 | yellow |
| SKA3 | 0.490989 | 4.35E-06 | yellow |
| TK1 | 0.490665 | 4.42E-06 | yellow |
| RAD51 | 0.489619 | 4.66E-06 | yellow |
| TYMS | 0.489402 | 4.72E-06 | yellow |
| MSTO1 | 0.488943 | 4.83E-06 | yellow |
| CENPL | 0.488784 | 4.87E-06 | yellow |
| SUV39H2 | 0.488465 | 4.95E-06 | yellow |
| MCM5 | 0.488301 | 4.99E-06 | yellow |
| CKAP2L | 0.486985 | 5.34E-06 | yellow |
| FAM72D | 0.485743 | 5.69E-06 | yellow |
| ZWINT | 0.485479 | 5.76E-06 | yellow |
| E2F2 | 0.484087 | 6.18E-06 | yellow |
| CBWD2 | 0.483691 | 6.31E-06 | yellow |
| RRM2 | 0.482727 | 6.62E-06 | yellow |
| C9orf110 | 0.481118 | 7.18E-06 | yellow |
| CDC25C | 0.480748 | 7.32E-06 | yellow |
| SGOL1 | 0.480407 | 7.44E-06 | yellow |
| DTL | 0.478772 | 8.07E-06 | yellow |
| CTSL2 | 0.478718 | 8.09E-06 | yellow |
| PBK | 0.476979 | 8.82E-06 | yellow |
| CNIH2 | 0.476826 | 8.89E-06 | yellow |
| DEPDC1 | 0.476424 | 9.07E-06 | yellow |
| PSRC1 | 0.475788 | 9.36E-06 | yellow |
| DBF4 | 0.474609 | 9.92E-06 | yellow |
| STIL | 0.474594 | 9.92E-06 | yellow |
| MYLK2 | 0.474368 | 1.00E-05 | yellow |
| HNRNPA2B1 | 0.473694 | 1.04E-05 | yellow |
| CDCA7 | 0.473488 | 1.05E-05 | yellow |
| TP73 | 0.472772 | 1.08E-05 | yellow |
| EPR1 | 0.471775 | 1.14E-05 | yellow |
| C11orf82 | 0.471715 | 1.14E-05 | yellow |
| BUB1B | 0.471448 | 1.16E-05 | yellow |
| TNFRSF13C | 0.470221 | 1.23E-05 | yellow |
| FAM111B | 0.470081 | 1.24E-05 | yellow |
| FBXO43 | 0.469754 | 1.26E-05 | yellow |
| DHFR | 0.469316 | 1.28E-05 | yellow |
| RAD54L | 0.468959 | 1.31E-05 | yellow |
| TPX2 | 0.467081 | 1.43E-05 | yellow |
| ERCC6L | 0.466587 | 1.46E-05 | yellow |
| FEN1 | 0.463888 | 1.66E-05 | yellow |
| ANP32B | 0.461706 | 1.84E-05 | yellow |
| ASF1B | 0.460243 | 1.98E-05 | yellow |
| OSTC | 0.460097 | 1.99E-05 | yellow |
| C13orf34 | 0.459978 | 2.00E-05 | yellow |
| WRAP53 | 0.45983 | 2.01E-05 | yellow |
| SPC24 | 0.459702 | 2.03E-05 | yellow |
| BIRC5 | 0.459104 | 2.08E-05 | yellow |
| TMEM194A | 0.458785 | 2.12E-05 | yellow |
| EZH2 | 0.457468 | 2.25E-05 | yellow |
| C9orf109 | 0.456771 | 2.32E-05 | yellow |
| NCAPH | 0.456738 | 2.33E-05 | yellow |
| POLD1 | 0.45593 | 2.42E-05 | yellow |
| SNRPD1 | 0.455268 | 2.49E-05 | yellow |
| MCM7 | 0.45433 | 2.60E-05 | yellow |
| EP400NL | 0.454275 | 2.61E-05 | yellow |
| NEIL3 | 0.452664 | 2.81E-05 | yellow |
| PPEF1 | 0.452033 | 2.89E-05 | yellow |
| RTKN2 | 0.451886 | 2.91E-05 | yellow |
| CENPE | 0.451752 | 2.93E-05 | yellow |
| C9orf140 | 0.451198 | 3.00E-05 | yellow |
| ARHGAP11A | 0.451156 | 3.01E-05 | yellow |
| FAM72A | 0.448471 | 3.40E-05 | yellow |
| RFC4 | 0.445315 | 3.92E-05 | yellow |
| FOXM1 | 0.445112 | 3.95E-05 | yellow |
| HMGB2 | 0.445061 | 3.96E-05 | yellow |
| KPNA2 | 0.444435 | 4.07E-05 | yellow |
| GPR137C | 0.444233 | 4.11E-05 | yellow |
| PLK4 | 0.443674 | 4.22E-05 | yellow |
| RIBC2 | 0.441946 | 4.55E-05 | yellow |
| MASTL | 0.441591 | 4.62E-05 | yellow |
| REEP4 | 0.440706 | 4.81E-05 | yellow |
| KNTC1 | 0.440684 | 4.81E-05 | yellow |
| CHAF1A | 0.44039 | 4.88E-05 | yellow |
| GINS2 | 0.440384 | 4.88E-05 | yellow |
| CBFB | 0.439739 | 5.02E-05 | yellow |
| TMSB15A | 0.439593 | 5.05E-05 | yellow |
| CENPK | 0.435906 | 5.93E-05 | yellow |
| CAD | 0.434518 | 6.30E-05 | yellow |
| NETO2 | 0.433934 | 6.46E-05 | yellow |
| CENPH | 0.433776 | 6.51E-05 | yellow |
| ORC1L | 0.432904 | 6.76E-05 | yellow |
| C15orf23 | 0.432662 | 6.83E-05 | yellow |
| FAM83D | 0.431008 | 7.33E-05 | yellow |
| FANCI | 0.428992 | 7.99E-05 | yellow |
| RACGAP1 | 0.427838 | 8.39E-05 | yellow |
| POLE2 | 0.425486 | 9.27E-05 | yellow |
| CEP152 | 0.424647 | 9.60E-05 | yellow |
| ZWILCH | 0.423834 | 9.93E-05 | yellow |
| RNASEH2A | 0.422188 | 0.000106 | yellow |
| MCM6 | 0.420221 | 0.000115 | yellow |
| CCDC18 | 0.418424 | 0.000124 | yellow |
| NMU | 0.418403 | 0.000124 | yellow |
| E2F7 | 0.418237 | 0.000125 | yellow |
| MYO19 | 0.417328 | 0.00013 | yellow |
| SKA1 | 0.417161 | 0.000131 | yellow |
| CDC6 | 0.416457 | 0.000135 | yellow |
| KIF20B | 0.416209 | 0.000136 | yellow |
| C18orf56 | 0.415971 | 0.000138 | yellow |
| CHTF18 | 0.415718 | 0.000139 | yellow |
| KIF15 | 0.414836 | 0.000144 | yellow |
| CDC25A | 0.414312 | 0.000147 | yellow |
| SLC35D3 | 0.412404 | 0.000159 | yellow |
| CDK2 | 0.411706 | 0.000163 | yellow |
| FANCD2 | 0.409905 | 0.000176 | yellow |
| LIN9 | 0.408798 | 0.000184 | yellow |
| CENPI | 0.408508 | 0.000186 | yellow |
| APOBEC3B | 0.407296 | 0.000195 | yellow |
| SGOL2 | 0.406004 | 0.000205 | yellow |
| C4orf46 | 0.405666 | 0.000208 | yellow |
| ACBD7 | 0.40542 | 0.00021 | yellow |
| NCAPD2 | 0.404279 | 0.00022 | yellow |
| CHAF1B | 0.403817 | 0.000224 | yellow |
| POLQ | 0.403252 | 0.000229 | yellow |
| ARL6IP6 | 0.401333 | 0.000247 | yellow |
| CCT6P1 | 0.40086 | 0.000251 | yellow |
| MAZ | 0.400649 | 0.000253 | yellow |
| SYCE2 | 0.400528 | 0.000255 | yellow |
| RAD51AP1 | 0.400005 | 0.00026 | yellow |
| SMC4 | 0.399976 | 0.00026 | yellow |
| ESCO2 | 0.39933 | 0.000267 | yellow |
| ESPL1 | 0.399205 | 0.000268 | yellow |
| ARHGAP11B | 0.398757 | 0.000273 | yellow |
| GINS4 | 0.398418 | 0.000276 | yellow |
| SFRS7 | 0.396095 | 0.000302 | yellow |
| SKA2 | 0.395718 | 0.000307 | yellow |
| ZNF367 | 0.395045 | 0.000315 | yellow |
| GLT25D1 | 0.39372 | 0.000331 | yellow |
| OIP5 | 0.393542 | 0.000333 | yellow |
| EFNA2 | 0.393397 | 0.000335 | yellow |
| NCAPG2 | 0.392 | 0.000353 | yellow |
| RFC3 | 0.391668 | 0.000358 | yellow |
| TCF19 | 0.390478 | 0.000374 | yellow |
| POLA2 | 0.390163 | 0.000379 | yellow |
| KIAA1524 | 0.389938 | 0.000382 | yellow |
| SETD8 | 0.389793 | 0.000384 | yellow |
| C21orf58 | 0.389645 | 0.000386 | yellow |
| ATAD2 | 0.388471 | 0.000403 | yellow |
| BRCA1 | 0.387781 | 0.000414 | yellow |
| MCM2 | 0.387756 | 0.000414 | yellow |
| CCNE2 | 0.387089 | 0.000425 | yellow |
| DTYMK | 0.386726 | 0.000431 | yellow |
| ANLN | 0.386513 | 0.000434 | yellow |
| ECT2 | 0.386373 | 0.000436 | yellow |
| POC1A | 0.38571 | 0.000447 | yellow |
| HMGA2 | 0.385466 | 0.000451 | yellow |
| SERPINH1 | 0.384829 | 0.000462 | yellow |
| DIAPH3 | 0.383842 | 0.000479 | yellow |
| CCDC15 | 0.382139 | 0.00051 | yellow |
| XRCC3 | 0.381454 | 0.000523 | yellow |
| XPO1 | 0.381054 | 0.000531 | yellow |
| FKBP10 | 0.380786 | 0.000536 | yellow |
| MYBL1 | 0.380148 | 0.000549 | yellow |
| XRCC2 | 0.379477 | 0.000563 | yellow |
| HIST2H2AB | 0.379228 | 0.000568 | yellow |
| C2orf48 | 0.378684 | 0.000579 | yellow |
| PYCR1 | 0.376094 | 0.000636 | yellow |
| C5orf34 | 0.375608 | 0.000647 | yellow |
| GPSM2 | 0.373669 | 0.000694 | yellow |
| SHCBP1 | 0.372678 | 0.000719 | yellow |
| CCDC150 | 0.372444 | 0.000725 | yellow |
| ZIC1 | 0.372232 | 0.00073 | yellow |
| C17orf53 | 0.37171 | 0.000744 | yellow |
| LOC81691 | 0.371674 | 0.000745 | yellow |
| HMGN2 | 0.371063 | 0.000761 | yellow |
| ATP2A1 | 0.36992 | 0.000793 | yellow |
| TMEM201 | 0.368298 | 0.000839 | yellow |
| ANP32E | 0.367363 | 0.000867 | yellow |
| RAD54B | 0.367308 | 0.000869 | yellow |
| DEPDC1B | 0.367209 | 0.000872 | yellow |
| CCDC77 | 0.36711 | 0.000875 | yellow |
| E2F1 | 0.366825 | 0.000884 | yellow |
| GPR153 | 0.365513 | 0.000925 | yellow |
| RDM1 | 0.364947 | 0.000943 | yellow |
| CHEK1 | 0.364414 | 0.000961 | yellow |
| NASP | 0.364265 | 0.000966 | yellow |
| DHX34 | 0.363645 | 0.000987 | yellow |
| FAP | 0.362003 | 0.001045 | yellow |
| GBX2 | 0.361415 | 0.001066 | yellow |
| RTTN | 0.360622 | 0.001095 | yellow |
| HIST1H3G | 0.359876 | 0.001124 | yellow |
| PLXNA1 | 0.359715 | 0.00113 | yellow |
| BLM | 0.358963 | 0.001159 | yellow |
| CENPW | 0.357399 | 0.001223 | yellow |
| TULP3 | 0.357037 | 0.001238 | yellow |
| GNB1L | 0.356703 | 0.001252 | yellow |
| SFRS3 | 0.355642 | 0.001298 | yellow |
| NCAPD3 | 0.354065 | 0.001368 | yellow |
| DCLRE1C | 0.353506 | 0.001394 | yellow |
| HN1L | 0.35327 | 0.001405 | yellow |
| DDN | 0.353013 | 0.001418 | yellow |
| C12orf48 | 0.352737 | 0.001431 | yellow |
| E2F8 | 0.351408 | 0.001496 | yellow |
| LOC339674 | 0.35048 | 0.001543 | yellow |
| DNMT1 | 0.349783 | 0.001579 | yellow |
| SLAMF9 | 0.349014 | 0.001619 | yellow |
| PPIL5 | 0.348792 | 0.001631 | yellow |
| FAM136A | 0.347519 | 0.001701 | yellow |
| PHGDH | 0.347025 | 0.001729 | yellow |
| ATAD5 | 0.346819 | 0.001741 | yellow |
| NFKBIL2 | 0.346307 | 0.00177 | yellow |
| THOC4 | 0.346101 | 0.001782 | yellow |
| TMEM105 | 0.345587 | 0.001812 | yellow |
| LIG1 | 0.345254 | 0.001832 | yellow |
| CDKN2BAS | 0.344 | 0.001908 | yellow |
| ORC6L | 0.343348 | 0.001949 | yellow |
| DOT1L | 0.34278 | 0.001985 | yellow |
| CCDC138 | 0.34231 | 0.002016 | yellow |
| CKAP2 | 0.341948 | 0.00204 | yellow |
| WDHD1 | 0.341457 | 0.002072 | yellow |
| FAT1 | 0.34119 | 0.00209 | yellow |
| CILP2 | 0.340268 | 0.002153 | yellow |
| TRAF2 | 0.337866 | 0.002325 | yellow |
| CCNF | 0.337681 | 0.002339 | yellow |
| GRM2 | 0.337252 | 0.002371 | yellow |
| APOBEC3A | 0.337128 | 0.00238 | yellow |
| ING1 | 0.337013 | 0.002389 | yellow |
| SMC1B | 0.336794 | 0.002406 | yellow |
| TRIM59 | 0.334257 | 0.002607 | yellow |
| DSN1 | 0.333883 | 0.002637 | yellow |
| CENPO | 0.333607 | 0.00266 | yellow |
| PI4KAP2 | 0.332374 | 0.002765 | yellow |
| FLVCR1 | 0.331754 | 0.002819 | yellow |
| HIST1H3B | 0.328627 | 0.003107 | yellow |
| MAD2L2 | 0.328201 | 0.003148 | yellow |
| RCE1 | 0.327938 | 0.003174 | yellow |
| QRFP | 0.327113 | 0.003256 | yellow |
| SFRS2 | 0.326193 | 0.003349 | yellow |
| WHSC1 | 0.324207 | 0.003559 | yellow |
| GINS1 | 0.323844 | 0.003598 | yellow |
| GPR3 | 0.323193 | 0.00367 | yellow |
| H2AFZ | 0.322431 | 0.003756 | yellow |
| C11orf24 | 0.322264 | 0.003775 | yellow |
| C4orf21 | 0.320947 | 0.003928 | yellow |
| DSCC1 | 0.319927 | 0.004051 | yellow |
| PHF13 | 0.319507 | 0.004102 | yellow |
| PBX4 | 0.317986 | 0.004293 | yellow |
| CEP110 | 0.317491 | 0.004357 | yellow |
| PXMP2 | 0.317171 | 0.004398 | yellow |
| MEX3D | 0.316781 | 0.00445 | yellow |
| INTS7 | 0.315164 | 0.004668 | yellow |
| C19orf76 | 0.315131 | 0.004673 | yellow |
| C19orf57 | 0.31508 | 0.00468 | yellow |
| SHOX2 | 0.313008 | 0.004974 | yellow |
| RFC2 | 0.311823 | 0.00515 | yellow |
| SUV39H1 | 0.311765 | 0.005159 | yellow |
| KPNB1 | 0.310531 | 0.005348 | yellow |
| SAAL1 | 0.310172 | 0.005404 | yellow |
| TET1 | 0.310017 | 0.005429 | yellow |
| GGH | 0.308375 | 0.005693 | yellow |
| HMMR | 0.308291 | 0.005707 | yellow |
| CASP3 | 0.308018 | 0.005752 | yellow |
| HIST1H2AJ | 0.307761 | 0.005795 | yellow |
| PPM1G | 0.307424 | 0.005852 | yellow |
| KDELR3 | 0.307283 | 0.005876 | yellow |
| NUSAP1 | 0.306438 | 0.00602 | yellow |
| LY6G6C | 0.306403 | 0.006026 | yellow |
| WDR76 | 0.305953 | 0.006105 | yellow |
| IL1F7 | 0.305732 | 0.006143 | yellow |
| CASC5 | 0.303653 | 0.006519 | yellow |
| CXCR7 | 0.302108 | 0.006811 | yellow |
| LBR | 0.302053 | 0.006822 | yellow |
| ZACN | 0.301939 | 0.006844 | yellow |
| C2orf77 | 0.301473 | 0.006935 | yellow |
| CSPG5 | 0.30133 | 0.006963 | yellow |
| MED8 | 0.300964 | 0.007035 | yellow |
| EPT1 | 0.30055 | 0.007118 | yellow |
| SASS6 | 0.300196 | 0.007189 | yellow |
| HAUS1 | 0.299903 | 0.007248 | yellow |
| FANCE | 0.299248 | 0.007383 | yellow |
| ADAM12 | 0.299225 | 0.007387 | yellow |
| TTK | 0.299056 | 0.007422 | yellow |
| SFPQ | 0.298212 | 0.0076 | yellow |
| ARHGAP33 | 0.298088 | 0.007626 | yellow |
| KIF4B | 0.297977 | 0.00765 | yellow |
| UBXN2A | 0.29764 | 0.007722 | yellow |
| NUDT1 | 0.297361 | 0.007782 | yellow |
| C1orf112 | 0.297013 | 0.007858 | yellow |
| RAB42 | 0.295969 | 0.008089 | yellow |
| HIST1H2AH | 0.295493 | 0.008197 | yellow |
| C13orf37 | 0.295157 | 0.008273 | yellow |
| CENPJ | 0.294785 | 0.008359 | yellow |
| YWHAH | 0.294529 | 0.008418 | yellow |
| TFDP1 | 0.293669 | 0.00862 | yellow |
| TOE1 | 0.293275 | 0.008714 | yellow |
| MCM3 | 0.292798 | 0.008829 | yellow |
| FBXO5 | 0.291962 | 0.009033 | yellow |
| ZDHHC18 | 0.291677 | 0.009104 | yellow |
| SKP2 | 0.291463 | 0.009157 | yellow |
| LMO7 | 0.291196 | 0.009224 | yellow |
| TTLL5 | 0.290421 | 0.009421 | yellow |
| COL5A1 | 0.289929 | 0.009548 | yellow |
| UAP1 | 0.289623 | 0.009627 | yellow |
| MNS1 | 0.289339 | 0.009702 | yellow |
| NOC2L | 0.289151 | 0.009751 | yellow |
| FAM54A | 0.287963 | 0.010069 | yellow |
| C1orf77 | 0.287922 | 0.010081 | yellow |
| EID3 | 0.287831 | 0.010105 | yellow |
| C12orf4 | 0.287779 | 0.01012 | yellow |
| HPDL | 0.287512 | 0.010193 | yellow |
| FAM64A | 0.28537 | 0.010796 | yellow |
| TBX1 | 0.284935 | 0.010922 | yellow |
| PLCXD1 | 0.284789 | 0.010965 | yellow |
| GNAI3 | 0.282615 | 0.011617 | yellow |
| DDX12 | 0.28241 | 0.01168 | yellow |
| USP1 | 0.281561 | 0.011945 | yellow |
| NID2 | 0.281261 | 0.01204 | yellow |
| PRR11 | 0.280791 | 0.01219 | yellow |
| C6orf167 | 0.279756 | 0.012526 | yellow |
| C1orf135 | 0.27926 | 0.01269 | yellow |
| CENPN | 0.278679 | 0.012884 | yellow |
| ZNF519 | 0.277401 | 0.013321 | yellow |
| POLE | 0.277066 | 0.013437 | yellow |
| PLOD1 | 0.275969 | 0.013825 | yellow |
| C1QTNF2 | 0.27554 | 0.013979 | yellow |
| MAD2L1 | 0.274328 | 0.014423 | yellow |
| RBM15 | 0.273157 | 0.014863 | yellow |
| RFC5 | 0.272493 | 0.015118 | yellow |
| RNASEH1 | 0.272409 | 0.015151 | yellow |
| KIF22 | 0.271955 | 0.015328 | yellow |
| LOC100128191 | 0.271891 | 0.015352 | yellow |
| CLSPN | 0.27174 | 0.015412 | yellow |
| CCDC99 | 0.270479 | 0.015914 | yellow |
| TGFBR1 | 0.270361 | 0.015962 | yellow |
| TUBB | 0.270308 | 0.015983 | yellow |
| CWF19L1 | 0.269446 | 0.016337 | yellow |
| BRIP1 | 0.26813 | 0.016888 | yellow |
| RNF138 | 0.267817 | 0.017022 | yellow |
| TUBB3 | 0.265442 | 0.018065 | yellow |
| DMC1 | 0.264407 | 0.018537 | yellow |
| GJC1 | 0.263506 | 0.018956 | yellow |
| LHX1 | 0.263441 | 0.018986 | yellow |
| PITX2 | 0.263417 | 0.018998 | yellow |
| POLD3 | 0.26276 | 0.019309 | yellow |
| WNT5A | 0.262711 | 0.019332 | yellow |
| HMGN1 | 0.262248 | 0.019555 | yellow |
| DBF4B | 0.261185 | 0.020074 | yellow |
| FANCB | 0.260376 | 0.020476 | yellow |
| RCC2 | 0.259686 | 0.020824 | yellow |
| SYNGR4 | 0.259429 | 0.020955 | yellow |
| LOC723972 | 0.259306 | 0.021019 | yellow |
| RAET1K | 0.256627 | 0.022432 | yellow |
| TAF5 | 0.256481 | 0.022511 | yellow |
| RACGAP1P | 0.256351 | 0.022582 | yellow |
| GMNN | 0.255363 | 0.023126 | yellow |
| EIF2C2 | 0.255255 | 0.023187 | yellow |
| FBXO46 | 0.255165 | 0.023237 | yellow |
| TTL | 0.253925 | 0.023939 | yellow |
| DCP2 | 0.253754 | 0.024037 | yellow |
| PASK | 0.253213 | 0.02435 | yellow |
| ZYG11A | 0.253016 | 0.024464 | yellow |
| LRRC59 | 0.251498 | 0.025365 | yellow |
| MYC | 0.251274 | 0.0255 | yellow |
| VRK1 | 0.250758 | 0.025813 | yellow |
| NRM | 0.249851 | 0.026373 | yellow |
| METTL10 | 0.249801 | 0.026404 | yellow |
| SMC2 | 0.249453 | 0.026622 | yellow |
| SLC35C1 | 0.248797 | 0.027036 | yellow |
| BRCA2 | 0.248137 | 0.027458 | yellow |
| RFWD3 | 0.247139 | 0.028107 | yellow |
| LOC642846 | 0.244553 | 0.029849 | yellow |
| C18orf54 | 0.243287 | 0.030735 | yellow |
| TUBA1B | 0.242746 | 0.03112 | yellow |
| TDG | 0.242515 | 0.031286 | yellow |
| NDE1 | 0.242409 | 0.031362 | yellow |
| EPHA3 | 0.242285 | 0.031451 | yellow |
| LOC100302401 | 0.242187 | 0.031522 | yellow |
| MMP9 | 0.24076 | 0.032569 | yellow |
| NPHP4 | 0.239112 | 0.033813 | yellow |
| GSTTP1 | 0.238273 | 0.034462 | yellow |
| CKM | 0.238197 | 0.034521 | yellow |
| SENP1 | 0.236742 | 0.035673 | yellow |
| SOX11 | 0.233264 | 0.038557 | yellow |
| HPCAL1 | 0.233127 | 0.038674 | yellow |
| SPIN4 | 0.232879 | 0.038888 | yellow |
| ICMT | 0.229906 | 0.041523 | yellow |
| GCHFR | 0.229377 | 0.042007 | yellow |
| TARDBP | 0.229129 | 0.042235 | yellow |
| ANGPTL3 | 0.226446 | 0.044774 | yellow |
| KIAA2013 | 0.226329 | 0.044888 | yellow |
| VGLL2 | 0.225179 | 0.046016 | yellow |
| HIST1H1E | 0.223693 | 0.04751 | yellow |
| NAV2 | 0.222748 | 0.04848 | yellow |
| PLK2 | 0.220972 | 0.050348 | yellow |
| NEURL1B | 0.220226 | 0.051149 | yellow |
| TIMELESS | 0.218532 | 0.053009 | yellow |
| DPH2 | 0.218136 | 0.053451 | yellow |
| MSH5 | 0.217769 | 0.053864 | yellow |
| KIAA1841 | 0.21688 | 0.054875 | yellow |
| CCDC19 | 0.216662 | 0.055125 | yellow |
| CTCFL | 0.216612 | 0.055183 | yellow |
| NRAS | 0.216272 | 0.055575 | yellow |
| IL8 | 0.215604 | 0.056353 | yellow |
| MCM4 | 0.214795 | 0.057307 | yellow |
| FANCA | 0.214659 | 0.057468 | yellow |
| PRIM1 | 0.214138 | 0.058091 | yellow |
| TMEM184B | 0.213823 | 0.058471 | yellow |
| LOC100130776 | 0.212659 | 0.059889 | yellow |
| GEN1 | 0.212467 | 0.060126 | yellow |
| FGFR1OP | 0.21163 | 0.061166 | yellow |
| PDSS1 | 0.210541 | 0.062542 | yellow |
| POLR1E | 0.21028 | 0.062874 | yellow |
| ATAD3A | 0.210183 | 0.062999 | yellow |
| EIF2AK2 | 0.209587 | 0.063768 | yellow |
| LOC401010 | 0.207548 | 0.066452 | yellow |
| PRPF38A | 0.206452 | 0.067932 | yellow |
| C17orf93 | 0.206282 | 0.068164 | yellow |
| RELT | 0.205612 | 0.069084 | yellow |
| HIST1H2BM | 0.204707 | 0.070344 | yellow |
| LYAR | 0.20461 | 0.07048 | yellow |
| IQCC | 0.204606 | 0.070485 | yellow |
| C1orf174 | 0.204516 | 0.070612 | yellow |
| WEE1 | 0.204449 | 0.070706 | yellow |
| TRAF7 | 0.202413 | 0.073618 | yellow |
| GPR113 | 0.201921 | 0.074337 | yellow |
| OAS3 | 0.200024 | 0.077157 | yellow |
| PHTF2 | 0.199987 | 0.077213 | yellow |
| LOC341056 | 0.19775 | 0.080652 | yellow |
| RELB | 0.19725 | 0.081436 | yellow |
| ACAN | 0.196992 | 0.081844 | yellow |
| C9orf69 | 0.196484 | 0.08265 | yellow |
| CA2 | 0.194899 | 0.085207 | yellow |
| ZNF695 | 0.194272 | 0.086238 | yellow |
| C13orf27 | 0.193657 | 0.087256 | yellow |
| C14orf145 | 0.193508 | 0.087504 | yellow |
| IKBIP | 0.191799 | 0.090392 | yellow |
| MCM8 | 0.191744 | 0.090485 | yellow |
| PGD | 0.190626 | 0.092417 | yellow |
| NUP35 | 0.190399 | 0.092813 | yellow |
| SMC6 | 0.190199 | 0.093163 | yellow |
| LASS5 | 0.190036 | 0.093449 | yellow |
| MAST2 | 0.189915 | 0.093662 | yellow |
| GALNT2 | 0.188796 | 0.095648 | yellow |
| PCNA | 0.188302 | 0.096536 | yellow |
| PPP1CC | 0.187139 | 0.098649 | yellow |
| HAUS7 | 0.187073 | 0.09877 | yellow |
| HNRNPM | 0.186822 | 0.099231 | yellow |
| MGC12982 | 0.186796 | 0.09928 | yellow |
| DDX11 | 0.186557 | 0.09972 | yellow |
| STX6 | 0.186335 | 0.100131 | yellow |
| LAMA1 | 0.186192 | 0.100397 | yellow |
| SPARC | 0.186138 | 0.100497 | yellow |
| YBX1 | 0.186128 | 0.100516 | yellow |
| BFAR | 0.185128 | 0.10239 | yellow |
| CDCA4 | 0.184351 | 0.103864 | yellow |
| CCDC21 | 0.1838 | 0.10492 | yellow |
| RAD18 | 0.183759 | 0.104999 | yellow |
| ALX3 | 0.183544 | 0.105412 | yellow |
| COL5A2 | 0.182081 | 0.108267 | yellow |
| RASSF1 | 0.179584 | 0.113276 | yellow |
| TPM4 | 0.176228 | 0.120288 | yellow |
| C15orf21 | 0.174827 | 0.123312 | yellow |
| TSEN54 | 0.174786 | 0.1234 | yellow |
| MND1 | 0.174208 | 0.124666 | yellow |
| EMILIN2 | 0.172735 | 0.127933 | yellow |
| SPSB4 | 0.172228 | 0.129075 | yellow |
| ACOT7 | 0.171802 | 0.130039 | yellow |
| HCG18 | 0.171794 | 0.130056 | yellow |
| GLYATL2 | 0.170925 | 0.132039 | yellow |
| MFI2 | 0.170354 | 0.133355 | yellow |
| HSPB11 | 0.168568 | 0.137536 | yellow |
| MMP1 | 0.167767 | 0.139442 | yellow |
| TFAP2A | 0.167288 | 0.14059 | yellow |
| OLFML2B | 0.166771 | 0.141839 | yellow |
| NFIL3 | 0.165397 | 0.145199 | yellow |
| CEP72 | 0.164824 | 0.146617 | yellow |
| ZBTB12 | 0.164786 | 0.146711 | yellow |
| SIP1 | 0.164439 | 0.147575 | yellow |
| TMTC3 | 0.164333 | 0.14784 | yellow |
| SAE1 | 0.163244 | 0.150583 | yellow |
| POLH | 0.162094 | 0.153518 | yellow |
| CDKN2A | 0.161289 | 0.155599 | yellow |
| EIF2C3 | 0.161119 | 0.15604 | yellow |
| ARPC1B | 0.160709 | 0.157109 | yellow |
| FJX1 | 0.16061 | 0.157369 | yellow |
| CYTH3 | 0.160399 | 0.157922 | yellow |
| LOC283867 | 0.159816 | 0.159457 | yellow |
| CSE1L | 0.159698 | 0.159771 | yellow |
| SNHG3-RCC1 | 0.156851 | 0.167445 | yellow |
| MTBP | 0.156728 | 0.167781 | yellow |
| TMEM106C | 0.154709 | 0.173393 | yellow |
| ZNF90 | 0.154041 | 0.175282 | yellow |
| PGM2L1 | 0.152397 | 0.179989 | yellow |
| VASH2 | 0.152088 | 0.180884 | yellow |
| DEM1 | 0.150955 | 0.184192 | yellow |
| DLEU2 | 0.149277 | 0.189175 | yellow |
| AKAP5 | 0.145541 | 0.200614 | yellow |
| PDIA4 | 0.145427 | 0.200971 | yellow |
| DCLRE1B | 0.144992 | 0.202337 | yellow |
| MTOR | 0.144779 | 0.203006 | yellow |
| SLITRK5 | 0.144426 | 0.204123 | yellow |
| C12orf76 | 0.143818 | 0.206053 | yellow |
| JAG1 | 0.143018 | 0.208616 | yellow |
| OASL | 0.142693 | 0.209661 | yellow |
| AK2 | 0.142186 | 0.211302 | yellow |
| KIAA0802 | 0.140783 | 0.21589 | yellow |
| NDRG1 | 0.139124 | 0.221404 | yellow |
| LOC144571 | 0.138854 | 0.222309 | yellow |
| CDCA7L | 0.138812 | 0.222451 | yellow |
| NUP93 | 0.138553 | 0.223323 | yellow |
| KIAA1609 | 0.135705 | 0.233075 | yellow |
| NRD1 | 0.133774 | 0.239855 | yellow |
| COL4A2 | 0.132179 | 0.245555 | yellow |
| FN1 | 0.131795 | 0.246942 | yellow |
| ZMYM1 | 0.129097 | 0.256835 | yellow |
| ARHGEF2 | 0.12896 | 0.257343 | yellow |
| HNRNPA1L2 | 0.128797 | 0.257952 | yellow |
| TDP1 | 0.125595 | 0.270075 | yellow |
| LRRC42 | 0.125453 | 0.27062 | yellow |
| SIX2 | 0.125247 | 0.271415 | yellow |
| DNAH8 | 0.123966 | 0.276386 | yellow |
| FANCM | 0.122863 | 0.280713 | yellow |
| TRAIP | 0.121966 | 0.284266 | yellow |
| LOC344967 | 0.121817 | 0.28486 | yellow |
| ADAMTS9 | 0.121079 | 0.287812 | yellow |
| BDKRB1 | 0.119598 | 0.293791 | yellow |
| TTC4 | 0.118052 | 0.300123 | yellow |
| FAM119B | 0.11798 | 0.300417 | yellow |
| GADD45A | 0.117965 | 0.300478 | yellow |
| COL3A1 | 0.117583 | 0.30206 | yellow |
| RPS10P7 | 0.116918 | 0.304819 | yellow |
| SLC30A7 | 0.115916 | 0.309014 | yellow |
| SHKBP1 | 0.115276 | 0.311707 | yellow |
| LYPD6 | 0.112078 | 0.325411 | yellow |
| LOC728643 | 0.111729 | 0.32693 | yellow |
| BRIX1 | 0.111056 | 0.32987 | yellow |
| C14orf106 | 0.109111 | 0.338465 | yellow |
| ZNF93 | 0.106695 | 0.349333 | yellow |
| ST6GALNAC4 | 0.104955 | 0.357291 | yellow |
| RASD1 | 0.104519 | 0.359305 | yellow |
| LYPLA2 | 0.103755 | 0.362845 | yellow |
| LOC399815 | 0.103721 | 0.363005 | yellow |
| BAX | 0.103416 | 0.364427 | yellow |
| TXNL4B | 0.102312 | 0.369594 | yellow |
| NSUN4 | 0.100426 | 0.378528 | yellow |
| C13orf38 | 0.100168 | 0.379762 | yellow |
| TMPO | 0.099714 | 0.381937 | yellow |
| KHDRBS1 | 0.09933 | 0.383783 | yellow |
| NLRP4 | 0.097741 | 0.391475 | yellow |
| RCC1 | 0.097313 | 0.39356 | yellow |
| LOXL2 | 0.095707 | 0.401454 | yellow |
| DNAJC8 | 0.095642 | 0.401773 | yellow |
| UQCRHL | 0.095448 | 0.402735 | yellow |
| SLC25A33 | 0.095376 | 0.40309 | yellow |
| ZNF652 | 0.094278 | 0.40855 | yellow |
| MYL6 | 0.093408 | 0.412907 | yellow |
| KDM1A | 0.09231 | 0.418445 | yellow |
| BDKRB2 | 0.091535 | 0.422378 | yellow |
| AREG | 0.091411 | 0.423012 | yellow |
| IL32 | 0.09043 | 0.428026 | yellow |
| SLC2A3 | 0.08969 | 0.431831 | yellow |
| C14orf143 | 0.088839 | 0.43623 | yellow |
| C6orf182 | 0.088587 | 0.43754 | yellow |
| HIST1H2AL | 0.087819 | 0.441539 | yellow |
| LRRC40 | 0.087546 | 0.442962 | yellow |
| PPIE | 0.086879 | 0.446459 | yellow |
| CASP7 | 0.086625 | 0.447797 | yellow |
| CDKN2C | 0.085669 | 0.452839 | yellow |
| WDR67 | 0.084967 | 0.456567 | yellow |
| MAPRE1 | 0.084891 | 0.456972 | yellow |
| FAM18B2 | 0.084602 | 0.458511 | yellow |
| EXOSC10 | 0.08459 | 0.458571 | yellow |
| ZNF833 | 0.083409 | 0.464894 | yellow |
| CAPZA1 | 0.083133 | 0.466377 | yellow |
| DENR | 0.081947 | 0.472784 | yellow |
| SLC1A1 | 0.080884 | 0.478561 | yellow |
| RHOC | 0.077948 | 0.494725 | yellow |
| COX4NB | 0.077692 | 0.496145 | yellow |
| LAMA4 | 0.077263 | 0.498534 | yellow |
| SLC4A5 | 0.074833 | 0.512176 | yellow |
| GLMN | 0.073859 | 0.517699 | yellow |
| PHTF1 | 0.073178 | 0.521578 | yellow |
| DRP2 | 0.072711 | 0.52425 | yellow |
| COL6A3 | 0.070956 | 0.534332 | yellow |
| RPSAP52 | 0.070913 | 0.534584 | yellow |
| PRIM2 | 0.070235 | 0.538504 | yellow |
| TEAD4 | 0.068966 | 0.545889 | yellow |
| ENO1 | 0.068838 | 0.546637 | yellow |
| RPA2 | 0.067905 | 0.552096 | yellow |
| FAM76A | 0.067279 | 0.555776 | yellow |
| NOL9 | 0.067003 | 0.557404 | yellow |
| DAXX | 0.065877 | 0.564059 | yellow |
| RNF2 | 0.063113 | 0.580559 | yellow |
| MYH9 | 0.06079 | 0.594591 | yellow |
| ERI3 | 0.0601 | 0.598789 | yellow |
| TXNDC12 | 0.059859 | 0.600262 | yellow |
| ANKRD13C | 0.059778 | 0.600751 | yellow |
| LOC286367 | 0.059289 | 0.603742 | yellow |
| LOC441601 | 0.057475 | 0.614879 | yellow |
| DNAJC11 | 0.055633 | 0.626279 | yellow |
| C3orf32 | 0.055611 | 0.626417 | yellow |
| CLIC1 | 0.054996 | 0.630241 | yellow |
| RALB | 0.053902 | 0.637072 | yellow |
| SERBP1 | 0.051494 | 0.652212 | yellow |
| HNRPDL | 0.051411 | 0.652734 | yellow |
| TEX15 | 0.049935 | 0.662089 | yellow |
| CEPT1 | 0.047975 | 0.674592 | yellow |
| PPP1R8 | 0.046902 | 0.681474 | yellow |
| SNORD1C | 0.046012 | 0.687198 | yellow |
| EDARADD | 0.045887 | 0.688005 | yellow |
| PPIH | 0.045592 | 0.689911 | yellow |
| LAMC1 | 0.045437 | 0.690907 | yellow |
| ACP6 | 0.044754 | 0.695328 | yellow |
| DNTTIP2 | 0.044683 | 0.695788 | yellow |
| CDC42 | 0.04291 | 0.707301 | yellow |
| JMJD6 | 0.042243 | 0.711645 | yellow |
| POLR3D | 0.040956 | 0.720065 | yellow |
| BZW2 | 0.04008 | 0.725815 | yellow |
| COPS8 | 0.039867 | 0.727213 | yellow |
| KCTD5 | 0.038873 | 0.733758 | yellow |
| MAN2C1 | 0.038273 | 0.737714 | yellow |
| SDS | 0.038262 | 0.737788 | yellow |
| TNFAIP8L1 | 0.037375 | 0.743657 | yellow |
| ACAD11 | 0.037189 | 0.744888 | yellow |
| GNG5 | 0.036025 | 0.752611 | yellow |
| YRDC | 0.035541 | 0.755833 | yellow |
| MTHFD2 | 0.034926 | 0.759927 | yellow |
| COL5A3 | 0.034327 | 0.763925 | yellow |
| PLXNA2 | 0.03429 | 0.764172 | yellow |
| ORAI1 | 0.033817 | 0.767333 | yellow |
| EPHA7 | 0.03378 | 0.767578 | yellow |
| AARS2 | 0.032692 | 0.774865 | yellow |
| UQCRH | 0.03259 | 0.775552 | yellow |
| SNRNP40 | 0.03217 | 0.778365 | yellow |
| CENPQ | 0.031364 | 0.783782 | yellow |
| ZMYM4 | 0.027054 | 0.81291 | yellow |
| RTCD1 | 0.026545 | 0.816368 | yellow |
| SF3A3 | 0.025001 | 0.826878 | yellow |
| RPF1 | 0.024614 | 0.829515 | yellow |
| LOC649330 | 0.023135 | 0.839623 | yellow |
| NECAP2 | 0.019948 | 0.861478 | yellow |
| ITGB3 | 0.019478 | 0.864712 | yellow |
| CREB5 | 0.018189 | 0.873585 | yellow |
| TTC23L | 0.017271 | 0.87992 | yellow |
| PDPN | 0.01674 | 0.883584 | yellow |
| COL4A1 | 0.016124 | 0.887842 | yellow |
| LYPD1 | 0.011895 | 0.917132 | yellow |
| TBX15 | 0.011138 | 0.922393 | yellow |
| LYPLA2P1 | 0.010198 | 0.928926 | yellow |
| VAMP2 | 0.009041 | 0.936969 | yellow |
| C10orf11 | 0.008793 | 0.938693 | yellow |
| JUN | 0.007941 | 0.944623 | yellow |
| GPR37L1 | 0.007842 | 0.945315 | yellow |
| GNB1 | 0.005977 | 0.958308 | yellow |
| NUP107 | 0.003475 | 0.97575 | yellow |
| BTF3L4 | 0.002064 | 0.985593 | yellow |
| C19orf69 | -0.00014 | 0.998992 | yellow |
| CYB5D1 | -0.00032 | 0.997764 | yellow |
| PPP2R5D | -0.00118 | 0.991791 | yellow |
| CNN3 | -0.00144 | 0.989975 | yellow |
| RBBP4 | -0.00148 | 0.989697 | yellow |
| ADAMTS4 | -0.00428 | 0.970172 | yellow |
| C1orf144 | -0.00432 | 0.969871 | yellow |
| ZCCHC17 | -0.00578 | 0.959691 | yellow |
| CALR3 | -0.00632 | 0.955884 | yellow |
| MYCBP | -0.00734 | 0.948791 | yellow |
| C12orf32 | -0.00895 | 0.937633 | yellow |
| FZD4 | -0.01156 | 0.919487 | yellow |
| RNF11 | -0.01169 | 0.918527 | yellow |
| C10orf62 | -0.01512 | 0.894781 | yellow |
| RIBC1 | -0.01614 | 0.88774 | yellow |
| INPP5K | -0.01623 | 0.88712 | yellow |
| LRRC41 | -0.01806 | 0.874506 | yellow |
| B4GALT6 | -0.0203 | 0.859086 | yellow |
| LOC643837 | -0.02234 | 0.845031 | yellow |
| SULT1A1 | -0.02697 | 0.81348 | yellow |
| PSMA5 | -0.02797 | 0.806701 | yellow |
| HSPA1L | -0.02981 | 0.794251 | yellow |
| METT10D | -0.03 | 0.792984 | yellow |
| WASF2 | -0.03114 | 0.785256 | yellow |
| GDF9 | -0.03204 | 0.779253 | yellow |
| GPRASP1 | -0.03206 | 0.779114 | yellow |
| BZRAP1 | -0.03244 | 0.776584 | yellow |
| ACCS | -0.0397 | 0.728326 | yellow |
| SLCO4C1 | -0.03984 | 0.727413 | yellow |
| CYP11B2 | -0.04143 | 0.716987 | yellow |
| DFFA | -0.04153 | 0.716332 | yellow |
| UBXN10 | -0.04213 | 0.712362 | yellow |
| CCT5 | -0.04259 | 0.709402 | yellow |
| ALDH3A2 | -0.04367 | 0.702364 | yellow |
| ANKRD2 | -0.04916 | 0.667032 | yellow |
| ZNF546 | -0.05156 | 0.651818 | yellow |
| C3orf18 | -0.05201 | 0.648933 | yellow |
| CMPK1 | -0.05556 | 0.62674 | yellow |
| DCAKD | -0.058 | 0.611626 | yellow |
| ASB14 | -0.0588 | 0.606748 | yellow |
| FBXO27 | -0.06024 | 0.597949 | yellow |
| PSMB2 | -0.06504 | 0.56902 | yellow |
| ABLIM1 | -0.06716 | 0.556474 | yellow |
| SIKE1 | -0.07033 | 0.53794 | yellow |
| ABCA5 | -0.07369 | 0.518643 | yellow |
| TMEM199 | -0.07937 | 0.486856 | yellow |
| TMC7 | -0.08452 | 0.458931 | yellow |
| C8orf42 | -0.08726 | 0.444437 | yellow |
| SLC4A9 | -0.08801 | 0.440538 | yellow |
| LAMB2 | -0.08848 | 0.43812 | yellow |
| SRC | -0.08887 | 0.436093 | yellow |
| AMZ2 | -0.08891 | 0.43585 | yellow |
| INADL | -0.09017 | 0.429362 | yellow |
| NDUFB4 | -0.0911 | 0.424615 | yellow |
| PLA2G2C | -0.0932 | 0.41393 | yellow |
| C7orf31 | -0.09388 | 0.41052 | yellow |
| CYYR1 | -0.09528 | 0.403579 | yellow |
| C17orf39 | -0.09603 | 0.399865 | yellow |
| NIPSNAP3B | -0.09675 | 0.396331 | yellow |
| CATSPER2 | -0.09974 | 0.381834 | yellow |
| C6orf70 | -0.0999 | 0.381038 | yellow |
| RBP4 | -0.104 | 0.36169 | yellow |
| ADCY2 | -0.10407 | 0.361373 | yellow |
| SLC26A1 | -0.10418 | 0.360868 | yellow |
| C6orf108 | -0.10534 | 0.355518 | yellow |
| ACOX1 | -0.10565 | 0.35411 | yellow |
| HDAC1 | -0.10642 | 0.35057 | yellow |
| C1orf51 | -0.1079 | 0.343883 | yellow |
| LOC100302640 | -0.10976 | 0.335573 | yellow |
| MRPL37 | -0.10996 | 0.334707 | yellow |
| DNAJC19 | -0.11033 | 0.333078 | yellow |
| SFRS2B | -0.11619 | 0.307861 | yellow |
| GLYCTK | -0.11707 | 0.304173 | yellow |
| ACOT6 | -0.11788 | 0.30083 | yellow |
| MXI1 | -0.1207 | 0.289321 | yellow |
| HLF | -0.12136 | 0.286699 | yellow |
| F10 | -0.12197 | 0.284265 | yellow |
| COX17 | -0.12199 | 0.284159 | yellow |
| ZNF238 | -0.12253 | 0.282044 | yellow |
| LOH12CR2 | -0.12428 | 0.275165 | yellow |
| BCL10 | -0.12458 | 0.273991 | yellow |
| LYNX1 | -0.12476 | 0.273294 | yellow |
| KCNH1 | -0.1267 | 0.265852 | yellow |
| TUSC2 | -0.13189 | 0.246604 | yellow |
| WDR5B | -0.13374 | 0.239964 | yellow |
| COLEC11 | -0.13485 | 0.236052 | yellow |
| RHOT1 | -0.13514 | 0.235053 | yellow |
| SLC37A1 | -0.13601 | 0.232025 | yellow |
| DNAJC28 | -0.13678 | 0.229374 | yellow |
| CCDC160 | -0.13901 | 0.221789 | yellow |
| HDAC5 | -0.143 | 0.208687 | yellow |
| P4HTM | -0.14403 | 0.205383 | yellow |
| PHACTR2 | -0.1458 | 0.199791 | yellow |
| KLHL10 | -0.14614 | 0.198761 | yellow |
| ACBD4 | -0.14722 | 0.195421 | yellow |
| NPM2 | -0.14725 | 0.195314 | yellow |
| PTP4A2 | -0.14756 | 0.194386 | yellow |
| PNRC1 | -0.14803 | 0.192936 | yellow |
| HDAC11 | -0.14831 | 0.192095 | yellow |
| SUGT1L1 | -0.15031 | 0.186095 | yellow |
| C12orf60 | -0.153 | 0.178252 | yellow |
| DIO3OS | -0.15475 | 0.173285 | yellow |
| CCBP2 | -0.15588 | 0.170121 | yellow |
| LOC678655 | -0.15589 | 0.170082 | yellow |
| ZBTB7B | -0.15821 | 0.163748 | yellow |
| C15orf34 | -0.15924 | 0.160995 | yellow |
| TSPAN31 | -0.15991 | 0.159216 | yellow |
| COQ10A | -0.16101 | 0.156326 | yellow |
| ANAPC13 | -0.16321 | 0.150657 | yellow |
| SHPK | -0.16368 | 0.149479 | yellow |
| BHMT | -0.16533 | 0.145366 | yellow |
| FADS6 | -0.16606 | 0.143577 | yellow |
| TRIM16L | -0.16728 | 0.140598 | yellow |
| CCDC28A | -0.16777 | 0.139435 | yellow |
| C11orf46 | -0.16828 | 0.138221 | yellow |
| MBNL2 | -0.16848 | 0.137752 | yellow |
| PPP1R9A | -0.16873 | 0.13715 | yellow |
| ZNF287 | -0.16967 | 0.134947 | yellow |
| MAP6 | -0.16979 | 0.134672 | yellow |
| NHEDC2 | -0.16994 | 0.134315 | yellow |
| LOC100130093 | -0.17326 | 0.126768 | yellow |
| DHFRL1 | -0.17474 | 0.123508 | yellow |
| CWC25 | -0.17511 | 0.122698 | yellow |
| NHEDC1 | -0.1761 | 0.12057 | yellow |
| TUBG2 | -0.17777 | 0.117031 | yellow |
| PCMTD2 | -0.18284 | 0.106787 | yellow |
| NHLH2 | -0.18333 | 0.105828 | yellow |
| GPN2 | -0.18609 | 0.100593 | yellow |
| L3MBTL4 | -0.18779 | 0.097461 | yellow |
| RARRES2 | -0.18909 | 0.095117 | yellow |
| AKNAD1 | -0.19047 | 0.092696 | yellow |
| C7orf10 | -0.19211 | 0.089866 | yellow |
| LRRC61 | -0.19232 | 0.089503 | yellow |
| KLF15 | -0.19647 | 0.082667 | yellow |
| CYP2C8 | -0.19728 | 0.081392 | yellow |
| ZNF396 | -0.19759 | 0.080899 | yellow |
| ADIPOR2 | -0.19765 | 0.080807 | yellow |
| SEC22B | -0.19823 | 0.079898 | yellow |
| BEND7 | -0.2006 | 0.076296 | yellow |
| STK33 | -0.20098 | 0.07573 | yellow |
| VPS18 | -0.20102 | 0.075664 | yellow |
| PLA2G16 | -0.20234 | 0.073721 | yellow |
| GHDC | -0.20462 | 0.070467 | yellow |
| DIRAS1 | -0.20491 | 0.070061 | yellow |
| FAM90A1 | -0.2052 | 0.069656 | yellow |
| SHISA4 | -0.20526 | 0.069569 | yellow |
| B3GNT1 | -0.20756 | 0.066438 | yellow |
| YPEL3 | -0.21006 | 0.063151 | yellow |
| PACSIN1 | -0.21021 | 0.062959 | yellow |
| PNPO | -0.21114 | 0.061786 | yellow |
| PFKFB1 | -0.21762 | 0.054037 | yellow |
| CBX7 | -0.22086 | 0.050469 | yellow |
| TBC1D4 | -0.22132 | 0.049976 | yellow |
| C17orf101 | -0.22306 | 0.048154 | yellow |
| TMEM116 | -0.22501 | 0.046186 | yellow |
| OXSM | -0.2279 | 0.043379 | yellow |
| RUFY3 | -0.22982 | 0.041598 | yellow |
| C10orf32 | -0.23363 | 0.038246 | yellow |
| MAP2K4 | -0.23523 | 0.036908 | yellow |
| C7orf29 | -0.23526 | 0.036882 | yellow |
| C2CD4A | -0.23978 | 0.033306 | yellow |
| EIF4E1B | -0.24418 | 0.030105 | yellow |
| OSBPL1A | -0.24457 | 0.02984 | yellow |
| TSPAN33 | -0.24498 | 0.029555 | yellow |
| SMARCD2 | -0.24499 | 0.029551 | yellow |
| SLC9A3R1 | -0.24517 | 0.029427 | yellow |
| KRT19 | -0.24668 | 0.028413 | yellow |
| LOC100128640 | -0.24737 | 0.027959 | yellow |
| KLHDC9 | -0.25206 | 0.02503 | yellow |
| AKT3 | -0.25208 | 0.025017 | yellow |
| ITFG2 | -0.25212 | 0.024994 | yellow |
| CYP4F12 | -0.25339 | 0.024247 | yellow |
| CDS1 | -0.25398 | 0.023908 | yellow |
| LOC644538 | -0.25482 | 0.023431 | yellow |
| CXorf58 | -0.25679 | 0.022344 | yellow |
| HHAT | -0.25797 | 0.021712 | yellow |
| CHAD | -0.25997 | 0.020683 | yellow |
| C9orf44 | -0.26267 | 0.01935 | yellow |
| ANKRD6 | -0.26361 | 0.018909 | yellow |
| DCP1B | -0.26662 | 0.017541 | yellow |
| DPH1 | -0.26664 | 0.017532 | yellow |
| PELI3 | -0.26844 | 0.016758 | yellow |
| RPS27L | -0.26854 | 0.016715 | yellow |
| HAGH | -0.27212 | 0.015264 | yellow |
| DTD1 | -0.27231 | 0.015188 | yellow |
| PION | -0.27277 | 0.01501 | yellow |
| THNSL1 | -0.27464 | 0.014309 | yellow |
| C2orf24 | -0.27468 | 0.014295 | yellow |
| ZNF117 | -0.27474 | 0.014271 | yellow |
| CRHBP | -0.27611 | 0.013774 | yellow |
| LOC149837 | -0.27646 | 0.013651 | yellow |
| LMOD1 | -0.27737 | 0.01333 | yellow |
| ABHD10 | -0.27808 | 0.013088 | yellow |
| C6orf225 | -0.2805 | 0.012285 | yellow |
| LOC255167 | -0.28454 | 0.011038 | yellow |
| PPAPDC3 | -0.28537 | 0.010796 | yellow |
| SPATA18 | -0.28631 | 0.010527 | yellow |
| KCNJ11 | -0.28801 | 0.010057 | yellow |
| ARHGEF10L | -0.29238 | 0.008931 | yellow |
| SERP2 | -0.29653 | 0.007965 | yellow |
| KIAA1143 | -0.29773 | 0.007703 | yellow |
| MEIS2 | -0.30094 | 0.00704 | yellow |
| ALAD | -0.30297 | 0.006646 | yellow |
| EIF4EBP3 | -0.30324 | 0.006595 | yellow |
| GPR98 | -0.30349 | 0.006549 | yellow |
| ARSD | -0.30404 | 0.006447 | yellow |
| AOX1 | -0.30559 | 0.006168 | yellow |
| UBR3 | -0.30892 | 0.005604 | yellow |
| KCNK15 | -0.3093 | 0.005542 | yellow |
| CYP7A1 | -0.30968 | 0.005482 | yellow |
| GPD1 | -0.30993 | 0.005443 | yellow |
| SH3BGR | -0.31065 | 0.005329 | yellow |
| CPEB1 | -0.31367 | 0.004879 | yellow |
| P2RX6 | -0.31523 | 0.004659 | yellow |
| C3orf23 | -0.31539 | 0.004637 | yellow |
| C14orf180 | -0.316 | 0.004555 | yellow |
| C2orf74 | -0.31616 | 0.004533 | yellow |
| RCBTB2 | -0.31657 | 0.004477 | yellow |
| CASD1 | -0.31955 | 0.004096 | yellow |
| LRRC8E | -0.32186 | 0.003822 | yellow |
| C17orf108 | -0.32231 | 0.003769 | yellow |
| EPHX2 | -0.32247 | 0.003751 | yellow |
| EXTL1 | -0.32387 | 0.003595 | yellow |
| HEBP2 | -0.3268 | 0.003288 | yellow |
| NUDT16P1 | -0.32867 | 0.003103 | yellow |
| RAG2 | -0.32968 | 0.003008 | yellow |
| SLITRK4 | -0.33062 | 0.002921 | yellow |
| CRBN | -0.33245 | 0.002759 | yellow |
| CCDC159 | -0.33633 | 0.002441 | yellow |
| ACTR3C | -0.34117 | 0.002092 | yellow |
| TNNI3K | -0.34302 | 0.00197 | yellow |
| MYRIP | -0.34835 | 0.001655 | yellow |
| PHF16 | -0.34909 | 0.001615 | yellow |
| ANKRD45 | -0.34956 | 0.00159 | yellow |
| THRSP | -0.35036 | 0.001549 | yellow |
| VPS52 | -0.35271 | 0.001432 | yellow |
| SLC25A38 | -0.35741 | 0.001222 | yellow |
| KNDC1 | -0.35764 | 0.001213 | yellow |
| TP53TG1 | -0.36377 | 0.000983 | yellow |
| FAM47E | -0.36404 | 0.000974 | yellow |
| DHDPSL | -0.36625 | 0.000902 | yellow |
| NDRG2 | -0.36802 | 0.000847 | yellow |
| CNGA1 | -0.37113 | 0.000759 | yellow |
| STOML1 | -0.37533 | 0.000654 | yellow |
| PLEKHM1 | -0.38065 | 0.000539 | yellow |
| SRGAP3 | -0.38239 | 0.000506 | yellow |
| MOCS1 | -0.38394 | 0.000478 | yellow |
| CYP4B1 | -0.38409 | 0.000475 | yellow |
| DUSP26 | -0.38779 | 0.000414 | yellow |
| NFKBIA | -0.38849 | 0.000403 | yellow |
| CCDC152 | -0.39465 | 0.000319 | yellow |
| PTGR2 | -0.39749 | 0.000286 | yellow |
| SEMA6A | -0.4096 | 0.000178 | yellow |
| SLC16A9 | -0.42207 | 0.000107 | yellow |
| ITM2B | -0.42216 | 0.000107 | yellow |
| TMEM140 | -0.42291 | 0.000103 | yellow |
| GNG7 | -0.4249 | 9.50E-05 | yellow |
| COLEC10 | -0.42914 | 7.94E-05 | yellow |
| C3 | -0.43129 | 7.24E-05 | yellow |
| PRNP | -0.44019 | 4.92E-05 | yellow |
| HPS1 | -0.44323 | 4.30E-05 | yellow |
| CRYL1 | -0.44706 | 3.62E-05 | yellow |
| FAM19A1 | -0.45585 | 2.42E-05 | yellow |
| C6orf217 | -0.45665 | 2.34E-05 | yellow |
| STAT5B | -0.47398 | 1.02E-05 | yellow |
| RNF180 | -0.50694 | 1.87E-06 | yellow |
| FBXO25 | -0.51615 | 1.12E-06 | yellow |
| CLYBL | -0.58897 | 1.13E-08 | yellow |
